# Decoding child speech in silence and noise: The type of background noise shapes adults’ processing

**DOI:** 10.3758/s13414-025-03194-4

**Published:** 2025-12-04

**Authors:** Marzie Samimifar, Federica Bulgarelli

**Affiliations:** 1 Department of Psychology, University at Buffalo – SUNY, 376 Park Hall, Buffalo, NY USA; 2 Department of Psychology, Department of Learning and Instruction, University at Buffalo – SUNY, 376 Park Hall, Buffalo, NY USA

**Keywords:** Child speech, Word recognition, Word prediction, Background noise, Eye-tracking

## Abstract

Processing speech that is non-canonical (i.e., child-produced speech) and/or presented in background noise can pose challenges for listeners. We investigated how listening to child-produced speech affects young adults’ word recognition under varying noise conditions. Participants (*n* = 121) completed a two-picture eye-tracking task in one of three conditions: no background noise, pink background noise, and real-world background noise from LENA recordings. Participants heard a child or adult (Speaker-Age) direct attention to a generic (e.g., keys) or child-specific (e.g., potty; Item-Type) item. We examined the effect of Speaker-Age and Item-Type on participants’ looking time. In no background noise, increases in target looking were high, with greater increases when adults produced generic items. Both pink noise and real-world noise increased task difficulty, but patterns of results varied as a function of speaker gender. For female speech, background noise resulted in an effect of Speaker-Age, with participants increasing their looking time more for adult relative to child speech. The type of background noise did not influence this pattern. For male speech, there was an effect of Speaker-Age in the opposite direction, with participants increasing their looking time more for child relative to adult speech. For male speech, real-world background noise resulted in higher increases in target looking for child-specific items. Together, results suggest that child-produced speech may be more difficult to process than female-adult produced speech in noise, and that listeners can use background noise to predict who will speak and what they might speak about under more challenging conditions, such as processing male speech.

**Supplementary Information:**

The online version contains supplementary material available at 10.3758/s13414-025-03194-4.

## Introduction

In everyday life, we hear speech from different people, and since each person sounds different, speech perception can be challenging. For example, the exact properties of speech vary as a function of the speaker’s age, sex, and language background (Boland et al., 2016). Environmental conditions, such as background noise, further complicate speech perception, leading to increased effort and potential errors in comprehension (Borghini & Hazan, 2020; Pichora-Fuller et al., 2016). One strategy listeners might use to overcome challenges posed by processing speech from different individuals in potentially noisy conditions is to engage in predictive processing, relying on their expectations about what specific speakers might say. Here, we are specifically interested in how young adults process speech from 5-year-old children (hereafter termed child-produced speech), whose speech has been shown to be less canonical and more challenging to comprehend (e.g. Yu et al. (2023)). We ask whether listeners use top-down information to facilitate or support processing of child-produced speech, and if so, what kinds of information listeners use. Specifically, we manipulate the type of items (child-specific vs. generic) and the background noise to test whether listeners use expectations about who is speaking and what they might be speaking about. By varying these factors, this set of studies advances our understanding of how listeners process non-canonical speech and how they may integrate expectations about speakers and background noise to aid in speech processing.

### Processing non-canonical speech

Previous research has found that non-canonical speech influences speech processing. A type of non-canonical speech that has received more attention is accented speech. While the exact definition of accented speech varies and can range from unfamiliar regional varieties of the native language to foreign-accented speech, processing accented speech appears to impair spoken word recognition (e.g., Lawson et al., 2003; Porretta et al., 2016). For example, Porretta et al. (2016) showed that looks to the target object in a visual-world eye-tracking paradigm decreased as the degree of foreign accentedness increased, suggesting that a stronger perceived foreign accent negatively impacted comprehension. Even regional accents can lead to processing costs, as for example, Canadian English speakers listening to British English were unable to rely on contrastive prosody to carry out a set of directions (Arnhold et al., 2020).

Here, we ask whether listening to child-produced speech also leads to processing difficulties. Children produce less canonical pronunciations of words, in part due to shorter vocal tracts and vocal folds, which result in a broader range of vowel durations compared to older children and adults, along with greater spectral and suprasegmental variations. Additionally, they exhibit more extensive variability in formant positions and fundamental frequencies within their speech signal (Benzeghiba et al., 2007), resulting in a generally higher pitch and longer segmental duration (Lee et al., 1999; Tingley & Allen, 1975). In addition, children often make predictable phonological errors (e.g. reduction of consonant clusters, ‘spaghetti’ becomes ‘paghetti’). Taken together, children’s developing articulatory and phonological skills result in variability and deviation in their pronunciation, pitch, and articulation.

Studies that have investigated adults’ processing of child-produced speech have found this speech to be more challenging. For example, Creel and Jimenez (2012) has shown that adults exhibit more difficulty learning to identify and recognize children’s voices. Similarly, Cooper et al. (2020) also found that adults exhibited difficulty even distinguishing between children’s voices, and were slower and less accurate at learning to identify individual child talkers relative to adult talkers. Children’s voices also appear to be harder to understand. In a recent study, adults were asked to transcribe single-word utterances from 2.5, 4, and 5.5-year-old children and adults. Adults were significantly more accurate at transcribing adult-produced speech relative to child-produced speech, though transcription accuracy improved as child age increased (Yu et al., 2023). Nonetheless, transcription accuracy for single-word utterances produced by 5.5-year-olds was 78%, compared to 87% accuracy for adult speech, suggesting that even 5-year-old children are more challenging to understand than adults.

### Effects of background noise

In addition to non-canonical speech potentially being more challenging to process, everyday environments often include background noise. Our daily experiences with language are often in less-than-ideal conditions due to potentially imperfect or degraded signals due to background noise (Borghini and Hazan (2020), see also Beaman (2005)), which in turn can make speech perception more effortful (Pichora-Fuller et al., 2016; Zekveld et al., 2014). For example, Strauß et al. (2022) found that increased background noise and decreased speech quality reduced perceptual clarity and led to greater uncertainty. Similarly, speed of word recognition was found to be slower and more effortful with both background noise and increased echoing (Picou et al., 2016).

Many of the studies described so far used more artificial background noise, such as pink or white noise, to test its effects on speech perception. However, while listeners in the real world do occasionally encounter these types of noises, they also typically encounter other kinds of background noises. A type of background noise commonly used is termed single or multi-talker babble, which consists of the target sound or sentence overlapped with sentences from one or multiple other talkers. While all noise seems to make listening comprehension more challenging, background noise that also contains speech appears to be the most challenging, possibly because it provides another stream that listeners could attend to (Eranović, 2022). Increasing the number of talkers in the background noise also further increases task difficulty (e.g Bronkhorst & Plomp 1992; Van Engen & Bradlow, 2007). Other types of more natural background noises have also been examined. For example, subway and vacuum noises (Lee et al., 2015) were found to affect participants’ ability to recognize words, though the individual effects varied. In sum, different types of background noise, whether artificial or naturally occurring, make speech perception more challenging, and background noise that contains speech is particularly difficult.

Background noise could be additionally challenging under already difficult processing conditions. For example, research suggests that the intelligibility of non-native speech declines more than native speech with the addition of different types of background noise, such as multi-talker babble (Rogers et al., 2006), speech-shaped noise (Bent & Atagi, 2015; Van Wijngaarden et al., 2002) and cafeteria noise (Munro, 1998). Thus, background noise could make processing child-produced speech even more challenging.

### The role of prediction

How might listeners overcome challenges posed by processing in these difficult contexts? One possibility is that listeners can rely on their expectations about upcoming speech. Previous research has found that listeners predict upcoming language as they encounter it, which can speed up processing when their predictions are correct (Pickering & Gambi, 2018). For example, highly predictive adjectives lead to preactivation of nouns (e.g., ‘soy’ preactivates ‘sauce, and ‘iced’ preactivates ‘tea’) (Fruchter et al., 2015). Furthermore, recognition of non-canonical word forms (e.g., phonological reductions) improves when they are preceded by a strongly supportive discourse context (Brouwer et al., 2013), suggesting that context can help listeners overcome challenging listening conditions. This has been found to extend to processing speech in noisy in addition to quiet conditions (Feest et al., 2019). In fact, prediction becomes especially helpful when listeners need to compensate for noisy input (Pickering & Garrod, 2007). Thus, listeners engage in prediction during speech processing, and can use it to optimize processing the speech signal, particularly in more challenging conditions.

In addition to predicting based on linguistic content, listeners can also predict based on non-linguistic properties, such as background knowledge or speaker intention. For example, Arnold et al. (2007) found that listeners looked at novel items more than familiar items when speakers produced disfluencies (e.g., ‘uh’; see also Bosker et al. (2014)). When disfluencies are produced by non-native speakers, however, listeners no longer interpret the disfluency to suggest that an unfamiliar object is being labeled (Bosker et al., 2014), suggesting that listeners incorporate the speaker’s perceived knowledge during speech processing. These types of predictions extend to speech produced by children. For example, adults who heard a child speaker say “every night I drink some wine before I go to bed” showed a larger N400 compared to when an adult produced the same sentence, indicating a mismatch in the listener’s expectations about upcoming words based on speaker (Van Berkum et al., 2008). Thus, listeners may have expectations about what types of words children will produce. Specifically for speech by or about children, previous research suggests that some words (e.g. potty, pacifier) are more associated with babies and children than others (e.g., car, keys; Perry et al. (2015)). If child-produced speech is more difficult to process, listeners may be aided by hearing children produce words that are associated with children. Recent modeling efforts support this idea, suggesting that adults engage in “child-directed listening”, listening with specific expectations regarding what children are likely to say and child-specific expectations about child pronunciations (Meylan et al., 2023), which allow them to interpret this otherwise noisy signal.

Additionally, it is possible that some types of background noise could actually be helpful for predicting in challenging listening environments. Gregg and Samuel (2009) suggest that auditory representations contain some semantic content (e.g., knowledge that the auditory input contained a dog barking and not a bell ringing), and these auditory representations could lead listeners to have expectations about the content of speech as well. Certain types of background noise may be particularly indicative of the presence of children (and therefore possibly discussion of child-specific things), which could allow listeners to switch to “child-directed listening” (Meylan et al., 2023), therefore aiding processing. Here, we investigate this possibility, testing speech processing in silence and in two types of background noise: speech-shaped pink noise and real-world background noise, asking whether different types of background noise differently impact speech processing.

**Table 1:** Characteristics of stimuli used in experiment. For both child-specific (left) and generic (right), the word, frequency rating, babiness rating, and proportion of 30-month-olds producing the word, from Perry et al. (2015)

Child-specific				Generic			
Word	Frequency	Babiness	30months%	Word	Frequency	Babiness	30months%
ball	1.55	6.00	100.0	zipper	0.60	2.70	80.00
bottle	1.08	9.60	97.5	glasses	1.59	1.10	81.30
block	0.98	4.47	92.5	horse	1.79	2.00	97.50
crayon	0.47	7.50	95.0	vacuum	0.96	2.00	83.80
kitty	1.10	4.10	97.5	fork	0.30	1.70	92.50
blanket	1.20	8.91	97.5	beads	0.47	2.63	48.80
balloon	0.55	6.54	100.0	hammer	0.86	4.90	76.30
diaper	1.11	6.91	96.3	chair	1.77	4.50	95.00
doll	0.94	7.13	90.0	bench	0.89	2.00	42.50
tummy	0.47	7.16	96.3	brush	1.08	2.30	92.50
cheerios	0.30	6.66	82.5	keys	0.87	3.36	42.50
potty	0.95	6.90	95.0	mop	0.60	2.25	58.80
Average	0.89	6.82	95.0	Average	0.95	2.62	74.29

### Current study

Taken together, processing non-canonical speech can be challenging for listeners, but listeners’ expectations or predictions about upcoming information could help them overcome these challenges. Here, we aim to specifically test how young adults process child-produced speech, investigating the role of prediction by manipulating the child-specificity of the target items, and the role of background noise by adding artificial (pink noise) and real-world (noise from children’s homes) background noise. We additionally collected self-reported data on participants’ experiences interacting with children, as prior experience with specific types of speech could improve comprehension (e.g., Yu et al., 2023; Bradlow & Bent 2008). We tested participants’ word recognition using a two-picture Visual World paradigm. We used a simple two-picture task so we could maximally compare the results with developmental populations in the future. We hypothesized that participants would be slower in looking and increase their target looking less overall when sentences were produced by a child speaker relative to an adult speaker, but that this effect may be less pronounced for child-specific words because child-specific items produced by children may facilitate prediction. Regarding the influence of background noise, we predict that the addition of pink background noise would increase the task difficulty overall, and that while real-world background noise may also be challenging for listeners, this type of noise may prime listeners to the presence of children, and thus the cost of processing child-produced speech may be reduced.

All experiments were preregistered on OSF: https://osf.io/ewphv/registrations.

## Experiment 1

In Experiment 1, we tested adults’ processing of adult-produced and child-produced speech in quiet, without any background noise. This serves as a baseline condition to test whether child-produced speech is more difficult to process.

### Method

#### Participants

Participants were 41 monolingual English speakers (mean age = 21, SD = 1.19). Participants self-disclosed their race and ethnicity: 21 identified as White, five as Asian, 12 as Black or African American, and three identified as Other; 39 identified as not Hispanic or Latino, and 2 were unknown or wished not to report. They were recruited from the University Subject Pool and received course credit for participation. A power analysis prior to data collection (see preregistration) determined that this sample size was sufficient to achieve .95 power with an estimated effect size of *f* = 0.25 and was consistent with previous research. To verify that they were monolingual, participants self-rated their proficiency in other languages on a ten-point scale, and none of them self-rated their proficiency in a language other than English as above a 6 (a criterion used to identify bilingual participants; Poepsel and Weiss (2016)). Informed consent was obtained from all participants prior to participation; all procedures were performed in compliance with the Declaration of Helsinki.

#### Stimuli

Stimuli were comprised of images (e.g., ball) and sentences labeling those images (e.g., “Look at the ball”). All stimuli are available on OSF: https://osf.io/ewphv/

**Fig. 1:**
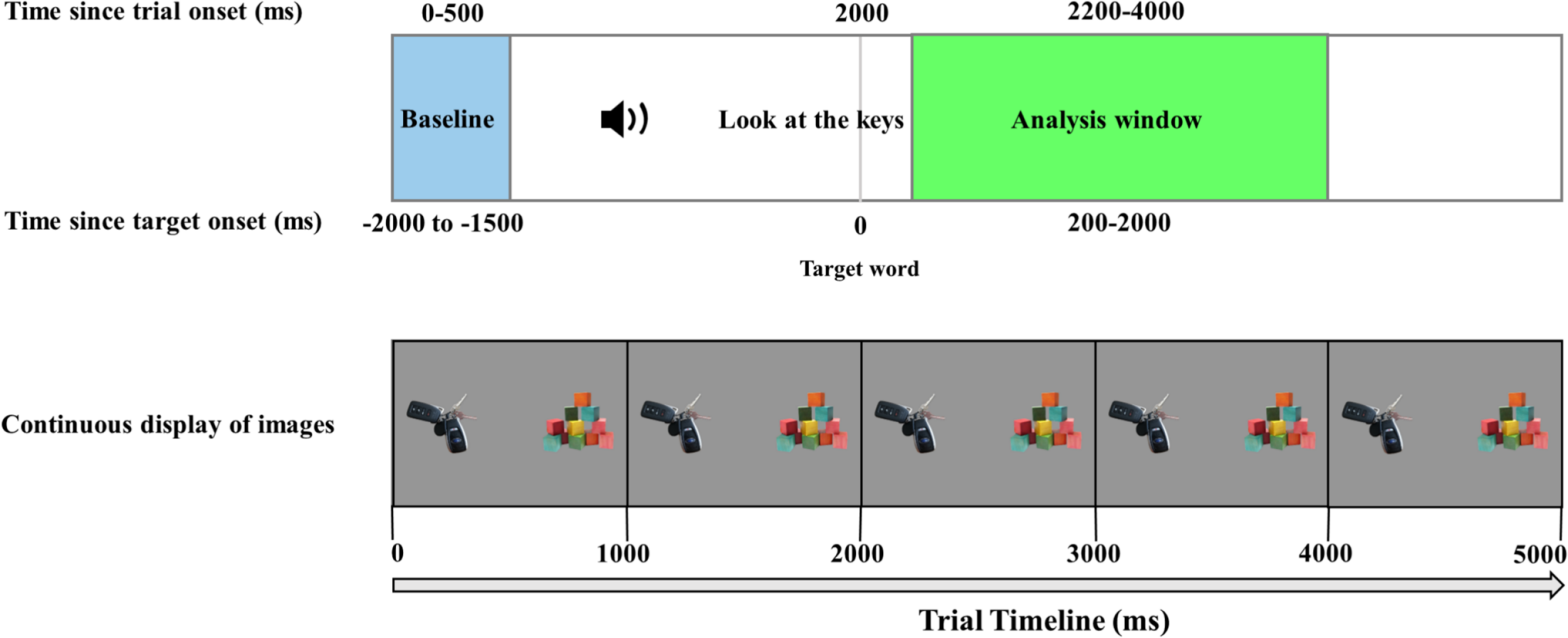
Set up of trials. The top timeline represents time since trial onset, the images were presented for 500 ms without any speech. Then, participants heard the directing sentence, telling them to look at one of the images, the target word occurred at 2000 ms. Below the timeline is time since target onset, with 0 now representing onset of the target word. The *light blue* portion represents time for baseline correcting, *green* represents the analysis window. Half of the trials (*n* = 24) were produced by an adult, and the other half (*n* = 24) by a child. In half of the trials, the target image was a child-specific item (e.g., blocks, blanket) and in the other half was a generic item (e.g., keys, hammer). Each trial lasted for 5000 ms

##### Visual stimuli

The visual stimuli consisted of 24 images. Half of the images were selected to have high child-specificity ratings (hereafter child-specific items) and the other half had low child-specificity ratings (hereafter generic items), as determined by Babiness ratings (see Perry et al. (2015); e.g., bottle and tummy are highly child-specific, fork and horse are generic; see Table 1). On each trial, two images were displayed, one child-specific and one generic. Each pair of images was yoked, such that they were always presented together. This allowed us to control for the effects of the distractor image on looking at the target image across trials. Images were edited in Photoshop and superimposed on a 500 x 500 pixel gray background.

##### Auditory stimuli

Auditory stimuli consisted of sentences labeling the target object embedded in carrier phrases directing participants to look at one of the images. The carrier phrases were “Look at the X”, “Can you find the X?”, “Where is the X?”, and “Do you see the X?” (see Bergelson and Swingley (2012)). The sentences were recorded from two parent–child pairs comprised of a same sex 5-year-old child and an adult (as at this age, transcription accuracy exceeds 50% but is still lower than accuracy for adult speech; Yu et al. (2023)). Specifically, one pair was a 5-year-old daughter and her mother, and the other pair was a 5-year-old son and his father. We recorded parent–child pairs to maximize the similarity of how specific phonemes were produced. Each target object was paired with a specific carrier phrase (e.g., can you find the car?), and speakers were asked to produce the full sentence. Both children had typical language development, and while they produced speech like children, they did not have any articulatory challenges (e.g., lisps, mispronunciations); see Supplemental Materials for analyses comparing mean pitch across speakers; all stimuli are available on OSF for readers to listen to. Stimuli were normalized to a peak intensity of 70.95 dB, and the timing of the target word was standardized so it always occurred 1500 ms into the sound file (and thus 2000 ms after trial onset, see below).

#### Procedure

##### Experimental task

The experimental task was a two-picture Visual World eye-tracking paradigm. After obtaining informed consent, participants were seated in a quiet, dimly lit room in front of a monitor (33.7 x 26.9cm screen, 1280 x 1024 resolution) equipped with an SR Research EyeLink 1000+ outfitted with a moveable arm. The eyetracker was set to “remote” mode, and it collected participants’ visual fixations, sampling monocularly at 500 Hz, using a small, high-contrast sticker on the participant’s forehead. Participants were first calibrated using a nine-point calibration. Then, they were told that they would see two pictures on the screen and hear one of them labeled and that they should follow the prompts they heard. Each test trial began with a fixation cross presented at the center of the screen for 500 ms followed by the presentation of two images positioned to the left and right of the screen for 500 ms. Following the presentation of images, they heard a sentence directing them to look at one of them (e.g., a doll and a vacuum and hearing “Where is the doll?”). The onset of the target word always occurred 2000 ms after trial onset, each trial lasted for 5000 ms. The schematic of the experimental trials is depicted in Fig. 1.

There were 48 trials in total. In half of the trials, the target image was child-specific (e.g. bottle, tummy), while the other half the target was a generic item (e.g. glasses, zipper). Also, half of the trials were produced by a child speaker, and half of the trials were produced by an adult. Speaker-Age was blocked such that each participant heard all the adult-produced or all the child-produced trials first, followed by all trials from the other speaker. Across participants, we counterbalanced speaker pair (father-son or mother-daughter), which Speaker-Age was heard first (adult first or child first), and trial order (to balance child-specific and generic items, and target side). We had eight total orders, and participants were randomly assigned to one of these eight orders. Therefore, Speaker-Age (adult vs. child) and Item-Type (child-specific vs. generic) were manipulated within subjects, while speaker pair, speaker order, and trial order were manipulated between subjects. Thus, each participant completed 12 trials for which the target word was child-specific, produced by a child speaker, 12 for which the target word was generic, produced by a child speaker, 12 for which the target word was child-specific, produced by an adult speaker, and 12 for which the target word was generic and produced by an adult speaker. The trial order within the Speaker-Age blocks was pseudo-randomized.

##### Questionnaires

After the experiment, participants completed two questionnaires. The first one was a Demographic Questionnaire that asked about their age, sex, race, and ethnicity as well as their language background, including all the languages they know, the age they started and stopped learning the language, and their proficiency in each language on a scale of 1–10. The second one was an Adult Exposure to Child Speech Questionnaire that asked about how frequently they interacted with children of different ages. For each age group, participants were asked to rate how frequently they interacted with kids of that age (daily, multiple times a week, weekly, monthly, yearly, or never), and in what capacity (e.g., their sibling, at a daycare job). Both questionnaires can be found on OSF.

#### Data analysis

We used R [Version 4.3.1; @] and the R-packages *}base* [@}R-base], *kableExtra* (Version 1.3.4; Zhu, 2021), *knitr* (Version 1.45; Xie, 2015), *papaja* (Version 0.1.1.9001; Aust & Barth, 2022), *readr* (Version 2.1.4; Wickham et al., 2023), and *tinylabels* (Version 0.2.4; Barth, 2023) for all our analyses and to generate this manuscript. Following the tutorial described in the eyetrackingR package (Forbes et al., 2023), we cleaned the eye-tracking data, calculated the amount of trackloss in each trial, and excluded any trial with over 25% trackloss.

To account for possible item idiosyncrasies or preferences, we first calculated baseline preference for the target on each trial by calculating the proportion of time participants looked at each image on the screen the first 500 ms of each trial (before any speech was heard). We chose this window of time because it was before participants heard individual speakers (adult or child) start producing the carrier phrase, which might on its own lead to predictive looking. We then corrected for these by-trial baseline preferences by subtracting the baseline preference from the looking time to the target, on a participant-by-trial basis. This method allows us to measure increases in looking time to the target after it has been named, controlling for whether participants were already more likely to look at the target on that trial.

We had two types of analyses; in the first analysis, we tested participants’ overall looking time across different trials. We used a linear mixed effects model to examine the influence of Speaker-Age (adult vs. child) and Item-Type (generic vs. child-specific) on corrected target looking. In our analyses, we selected the adult speaker as the reference category for the Speaker-Age variable and the generic Item-Type as the reference category for the Item-Type variable. This allowed us to compare the effects of child speakers and child-specific items relative to these baselines.

CorrectedProportionTargetLooking = ProportionTargetLooking (between 200 and 2000 ms post target onset) - BaselineTargetLooking (between 0 and 500 ms from trial onset)

In the second analysis, we employed a growth curve modeling approach following Mirman (2017) to test whether the looking trajectory to the target object differed as a function of (1) whether the target was named by an adult or a child (Speaker-Age) and (2) whether the word was considered to be child-specific or not (Item-Type). Here, too, we accounted for baseline preferences by subtracting the baseline preference from each time bin (see below) for the growth curve analysis.

Across all three experiments, the data were binned into 20-ms time intervals, and the proportions of target fixation for each Item-Type were analyzed from 200 to 2000 ms post-word onset (1800 ms total, see preregistration). We considered Speaker-Age and Item-Type as predictors. We fit a series of mixed-effects models to predict participants’ looking to the target based on the condition of each trial while accounting for random intercepts across trials and subjects. The linear mixed-effects models were fitted using the lmer function from the lme4 package in R. We started the model selection process with a model that included only a linear time term. Then, step by step, we added quadratic, cubic and quartic time terms respectively to assess if they provided a better fit to the data. Each time, we compared the fit of the new model to the previous one using an ANOVA. Finding that the new model provided a significantly better fit, we proceeded by adding another time term. This stepwise approach allowed us to determine the most appropriate model. Additionally, all of the models incorporated interactions between Speaker-Age, Item-Type and each time term, allowing us to assess the impact of these variables over time. Also, to account for variability across different trials and subjects, we included by item and by subject random intercepts.

### Results

At the first step of data preprocessing (before using the corrected proportion of target looking), we tested for the percentage of track loss at the trial level for each participant and excluded any trial that had> 25% track loss. This resulted in the removal of 56 trials from 18 participants. On average, participants contributed data from 47 trials, ranging from 37 to 48 trials each. All participants contributed a minimum of 18 trials in the adult speaker condition and 18 trials in the child speaker condition, meeting our preregistered criteria for inclusion.

Then, using our corrected proportion of target looking, we also checked whether aspects of our counterbalancing (i.e., whether the first speaker was an adult vs. child, and whether they were in the male speaker vs. female speaker condition) influenced overall performance. The age of the first speaker ($b = 0.00$, 95% CI $[-0.04, 0.03]$, $\textit{t} (38) = -0.24$, $\textit{p} = .812$) did not influence participants’ overall performance during the task, but speaker gender did ($b = 0.04$, 95% CI $[0.00, 0.07]$, $\textit{t} (38) = 2.24$, $\textit{p} = .031$), such that participants increased their looking time relative to baseline more when hearing male speech (M = 44.70, SD = 4.75) than when hearing female speech (M = 40.90, SD = 5.98).

Our preregistered analysis plan was to test whether overall increases in target looking differed by (1) Speaker-Age (child or adult) (2) Item-Type (generic or child-specific), and (3) the interaction between the two. Because we found a significant difference in target looking for male vs. female speech, we also ran a model that included speaker-gender, allowing it to interact with Speaker-Age and Item-Type, and tested whether this was a better fit for the data.

The model with speaker-gender was not a significantly better fit (p = .166), and therefore we interpret the preregistered model without speaker gender. The effect of Speaker-Age ($\hat{\beta } = -0.01$, 95% CI $[-0.03, 0.00]$, $\textit{t} (117) = -1.65$, $\textit{p} = .102$) and the effect of Item-Type ($\hat{\beta } = 0.00$, 95% CI $[-0.02, 0.01]$, $\textit{t} (117) = -0.14$, $\textit{p} = .886$) were not significant. However, the interaction between Speaker-Age and Item-Type was significant ($\hat{\beta } = 0.02$, 95% CI $[0.00, 0.03]$, $\textit{t} (117) = 2.00$, $\textit{p} = .048$), such that participants increased their looking time significantly more to generic items produced by adults (M = 45.80) relative to children (M = 40.29, $\textit{t} (77.23) = 2.29$, $\textit{p} = .025$), but did not differ in their increase in looking time to specific items across speakers ($\textit{t} (75.60) = -0.26$, $\textit{p} = .799$; M~Child Specific~ = 43.09, M~Adult Specific~ = 42.56). Overall, this analysis suggests that participants spend more time looking at the target when the target is a generic item produced by adults, and spend the least time looking when children produce generic items. However, listening to children produce child-specific words did not result in larger increases in target looking. Together, this suggests that participants are potentially integrating speaker information with the referent, though we do not see the expected facilitation when children are producing child-specific items.

**Table 2:** Experiment 1 Growth curve modeling results for female speech condition. Ot1 refers to linear term, Ot2 refers to quadratic term, Ot3 refers to cubic term, Ot4 refers to quartic term

Term	$\hat{\beta }$	95% CI	*t*	$\textit{df}$	*p*
Intercept	0.37	[0.33, 0.41]	17.41	38.27	< .001
Ot1	0.89	[0.86, 0.93]	53.78	74,370.95	< .001
Ot2	-0.52	[-0.55, -0.49]	-31.26	74,370.95	< .001
Ot3	0.18	[0.14, 0.21]	10.68	74,370.95	< .001
Ot4	-0.05	[-0.08, -0.02]	-2.95	74,370.95	.003
Speaker contrast1	-0.01	[-0.01, -0.01]	-6.06	74,375.76	< .001
Type contrast1	0.00	[-0.03, 0.03]	-0.02	22.00	.984
Ot1 $\times$ Speaker contrast1	0.05	[0.01, 0.08]	2.79	74,370.95	.005
Ot2 $\times$ Speaker contrast1	0.02	[-0.01, 0.05]	1.24	74,370.95	.215
Ot3 $\times$ Speaker contrast1	-0.01	[-0.04, 0.02]	-0.50	74,370.95	.615
Ot4 $\times$ Speaker contrast1	0.00	[-0.04, 0.03]	-0.24	74,370.95	.808
Ot1 $\times$ Type contrast1	0.09	[0.06, 0.12]	5.55	74,370.95	< .001
Ot2 $\times$ Type contrast1	-0.07	[-0.10, -0.04]	-4.32	74,370.95	< .001
Ot3 $\times$ Type contrast1	0.03	[0.00, 0.06]	1.82	74,370.95	.069
Ot4 $\times$ Type contrast1	0.00	[-0.04, 0.03]	-0.17	74,370.95	.864
Speaker contrast1 $\times$ Type contrast1	0.01	[0.00, 0.01]	4.64	74,374.35	< .001
Ot1 $\times$ Speaker contrast1 $\times$ Type contrast1	0.03	[0.00, 0.07]	2.02	74,370.95	.043
Ot2 $\times$ Speaker contrast1 $\times$ Type contrast1	-0.05	[-0.09, -0.02]	-3.16	74,370.95	.002
Ot3 $\times$ Speaker contrast1 $\times$ Type contrast1	0.04	[0.01, 0.07]	2.48	74,370.95	.013
Ot4 $\times$ Speaker contrast1 $\times$ Type contrast1	-0.02	[-0.06, 0.01]	-1.36	74,370.95	.173

**Fig. 2:**
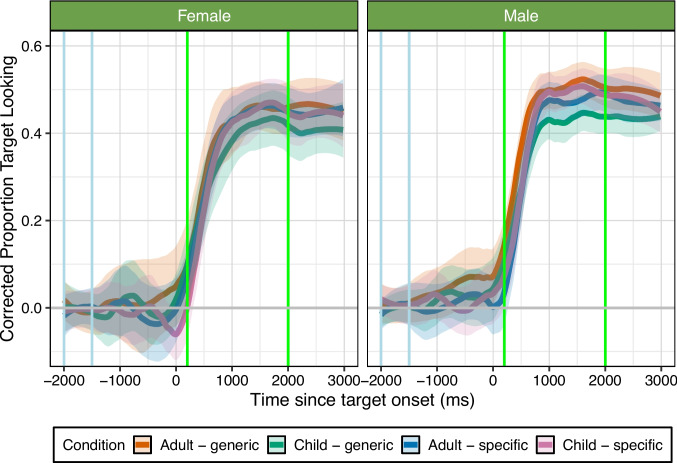
Experiment 1: corrected proportion of looking to the target over time based on condition. Each *line* represents one Speaker-Age and Item-Type condition mean, and shaded ribbons show 95% confidence intervals using a normal distribution, smoothed using a span of 0.25. The *x*-axis shows the time since word onset in milliseconds (ms) and the *y*-axis indicates the proportion of target looking; -2000 to -1500 ms (blue vertical lines) is the baseline preference window of analysis, target word happened at 0 ms, 200–2000ms (green vertical lines) is the time window for analysis. The *grey horizontal line* indicates 0 change from baseline looking. Overall, this graph shows the highest looking to the target when the Speaker-Age and Item-Type were congruent. See Supplementals for looking time graph with overlaid model predictions

In the next analysis, we consider the looking time over the trial using a growth-curve model. This allows us to test whether the trajectory of looking time differs across these conditions. We followed the best practices outlined in the tutorial for eyetrackingR (Dink & Ferguson, 2015; Mirman, 2017) for these growth curve models. A model with main effects of Speaker-Age, Item-Type, and interactions with four time terms (linear, quadratic, cubic, and quartic) was a better fit than models with three time terms or less (*p* < .001). Here, too, we tested whether adding speaker-gender improved model fit. The model that included speaker-gender and all its interactions was a significantly better fit for the data (*p* < .001). This model included a significant four-way interaction between the cubic time term and Speaker-Age, Item-Type, and speaker gender. Since four-way interactions are incredibly difficult to interpret, we re-ran the growth curve models for each speaker-gender condition separately.

In the female speaker condition, there was a significant interaction between Speaker-Age and Item-Type, as well as significant three-way interactions between the linear, quadratic, and cubic terms and Speaker-Age and Item-Type, see Table 2. Figure 2 shows that participants increased their looking time faster (linear term) and reached different peaks (quadratic term) and different peak sharpness (cubic term) across different conditions, with the highest target looking for item types that were congruent with the speaker: adults producing generic items and children producing child-specific items. They increased their looking time least when children produced generic items.

In the male speaker condition, in contrast, there were no significant three-way interactions, but there were significant interactions between the cubic and quartic time terms and Speaker-Age, and significant interactions between all four time terms and Item-Type, see Table 3. Figure 2 shows similar overall patterns of target looking relative to the female speaker conditions. However, the steepness (linear term) of increases in looking time and the peak (quadratic term) varied only as a function of Item-Type, with participants reaching higher peaks faster for adult relative to child-produced speech, while peak sharpness and shape (cubic and quartic terms) differing for both Speaker-Age and Item-Type. More specifically, participants achieved the highest target looking again for item types that were congruent with the speaker, when adults produced generic items and children produced child-specific items. Participants also reached a higher peak than they did in the female speech condition. Here, too, the target looking for children producing generic items was lowest.

#### Exploratory analysis of self-reported data on experience with children

While increases in target looking were high overall, it is possible that target looking varied across participants based on their own experiences interacting with children. To investigate this question, we analyzed participants’ self-reported data about their frequency of interaction with children. Participants were asked to rate how often they interact with children across five different age groups: under 1 year, 1–2 years, 3–4 years, 5–6 years, and 7 years or older. The scale of responses included: ‘Never,’ ‘Yearly (once a year),’ ‘Monthly (once a month),’ ‘Weekly (once a week),’ ‘Multiple times a week,’ and ‘Daily.’ Each response was mapped to a numerical score from 0 to 5 respectively (‘Never’ = 0, ‘Yearly (once a year)’ = 1, ‘Monthly (once a month)’ = 2, ‘Weekly (once a week)’ = 3, ‘Multiple times a week’ = 4, ‘Daily’ = 5) for each of the five age groups. Next, we generated an “Experience with Children Score” that reflected each participant’s overall frequency of experience with children. In principle, the possible range of scores is 0 (reflecting never interacting with children in any of the age groups) to 20 (reflecting daily interaction with children in each age group). In this group of participants, the range of responses was 0–17, with a mean of 6.62, suggesting relatively little experience with children overall. We added this Experience with Children Score to our best-fit model that included Speaker-Age, Item-Type, speaker gender, four time-terms, and the interaction between them as the predictors. Then, we used ANOVA to compare the previous best-fit model (with four time terms and speaker gender) with the one including Experience with Children Score and saw that the addition of this score did not significantly improve model fit (*p* = .065), see Supplemental Materials for histograms plotting the distribution of these scores as well as additional analyses using a categorical variable of experience.

**Table 3:** Experiment 1 growth curve modeling results for male speech condition. Ot1 refers to linear term, Ot2 referes to quadratic term, Ot3 refers to cubic term, Ot4 refers to quartic term

Term	$\hat{\beta }$	95% CI	*t*	$\textit{df}$	*p*
Intercept	0.41	[0.38, 0.44]	25.87	40.25	< .001
Ot1	0.88	[0.86, 0.91]	60.30	93,267.00	< .001
Ot2	-0.63	[-0.66, -0.60]	-43.07	93,267.00	< .001
Ot3	0.28	[0.25, 0.31]	18.87	93,267.00	< .001
Ot4	-0.07	[-0.09, -0.04]	-4.45	93,267.00	< .001
Speaker contrast1	-0.01	[-0.02, -0.01]	-9.56	93,272.23	< .001
Type contrast1	0.00	[-0.03, 0.02]	-0.30	22.00	.768
Ot1 $\times$ Speaker contrast1	-0.01	[-0.03, 0.02]	-0.37	93,267.00	.709
Ot2 $\times$ Speaker contrast1	0.00	[-0.03, 0.03]	0.05	93,267.00	.961
Ot3 $\times$ Speaker contrast1	-0.05	[-0.08, -0.02]	-3.64	93,267.00	< .001
Ot4 $\times$ Speaker contrast1	0.03	[0.00, 0.06]	2.24	93,267.00	.025
Ot1 $\times$ Type contrast1	0.13	[0.10, 0.16]	8.83	93,267.00	< .001
Ot2 $\times$ Type contrast1	-0.09	[-0.12, -0.06]	-6.13	93,267.00	< .001
Ot3 $\times$ Type contrast1	0.03	[0.01, 0.06]	2.38	93,267.00	.017
Ot4 $\times$ Type contrast1	0.04	[0.01, 0.07]	2.62	93,267.00	.009
Speaker contrast1 $\times$ Type contrast1	0.02	[0.02, 0.03]	15.22	93,268.82	< .001
Ot1 $\times$ Speaker contrast1 $\times$ Type contrast1	0.00	[-0.03, 0.02]	-0.29	93,267.00	.773
Ot2 $\times$ Speaker contrast1 $\times$ Type contrast1	-0.02	[-0.05, 0.01]	-1.38	93,267.00	.167
Ot3 $\times$ Speaker contrast1 $\times$ Type contrast1	-0.01	[-0.04, 0.02]	-0.52	93,267.00	.606
Ot4 $\times$ Speaker contrast1 $\times$ Type contrast1	-0.02	[-0.05, 0.01]	-1.18	93,267.00	.239

### Experiment 1 Discussion

In brief, we found that this task was relatively easy, with participants significantly increasing their target looking across conditions in this simple two-picture Visual World task. We hypothesized that speech from children might be more challenging to process, but we did not find any overall effect of Speaker-Age. We also hypothesized that processing would be easier when there was a ‘match’ between the speaker and the item, such that generic items produced by adults and child-specific items produced by children would be easiest for participants to predict and respond to. However, we only found evidence for one half of this hypothesis, across model types, participants seemed to increase their looking time most when adults produced generic items, and least when children produced generic items, but we did not find evidence for an advantage in processing child-specific items produced by children. Together, we take these results to cautiously suggest that participants are integrating information about the speaker when creating expectations about what they might say. In Experiment 2, we add pink speech-shaped background noise to test processing of child and adult speech, and the role of prediction about what speakers might say, under more difficult conditions.

## Experiment 2

### Method

#### Participants

Forty-one monolingual English speakers (mean age = 20, SD = 1.94) participated in Experiment 2. They self-disclosed their race and ethnicity: 29 identified as White, 3 as Asian, 6 as Black or African American, and three identified as Other; 38 identified as not Hispanic or Latino, two Hispanic or Latino, and one was unknown or wished not to report.

#### Stimuli

Materials used in this experiment were the same as in Experiment 1 except for the presence of pink background noise. We generated the pink noise using the Praat Vocal Toolkit (Corretge, 2012), which uses a Gaussian distribution (mean = 0, standard deviation = 1) with a sampling rate of 44,100, and has equal spectral power per frequency bin on a logarithmic frequency scale. We measured the amplitude of each individual carrier phrase and matched the amplitude of the pink noise so that each file had a 0dB signal-to-noise ratio. The pink noise was matched in length with the entire spoken utterance, such that the noise only occurred during the speech signal and participants could not habituate to it prior to speech onset. We chose to use pink noise because it is speech-shaped background noise (in contrast to white noise), and has been shown to increase speech perception difficulty, but does not include other types of noises that may further increase difficulty, compete for attention, or potentially provide information about upcoming speech (e.g., multi-talker babble, other naturalistic markers) (Maillard et al., 2023). Also, compared to white noise, pink noise is significantly less unpleasant and induces lower feelings of unease, which makes it a more suitable energetic masker in experimental contexts (Færøvik et al., 2025). As in Experiment 1, silence was added so that the target word always occurred 2000 ms after trial onset.

#### Procedure

We used the same procedure as in Experiment 1.

#### Data analysis

Data analysis followed the same protocol as in Experiment 1.

### Results

As above, we first preprocessed the data; 56 trials from 13 participants were excluded because of having more than 25% track loss. The minimum number of trials contributed by all of the participants was 19 in the adult-speaker condition and 15 in the child-speaker condition. They all contributed data from 47 trials on average, ranging from 34 to 48.

We then used our corrected proportion target looking to test for the influence of our counterbalancing choices (i.e., whether the first speaker was an adult vs. child, and whether they were in the male speaker vs. female speaker condition) on participants’ performance. Age of the first speaker did not influence participants’ performance during the task ($b = -0.01$, 95% CI $[-0.03, 0.01]$, $\textit{t}(39) = -1.05$, $\textit{p} = .302$), but speaker gender did ($b = 0.03$, 95% CI $[0.01, 0.05]$, $\textit{t}(39) = 2.84$, $\textit{p} = .007$) such that participants increased their target looking more for female speakers (M = 38.83, SD = 5) than male speakers (M = 33.06, SD = 7).

Then, we analyzed the overall increases in target looking as a function of the Speaker-Age, Item-Type, and their interaction. As for Experiment 1, we tested whether adding speaker-gender improved model fit. The model with speaker-gender and its interactions was a significantly better fit for the data (*p* < .001), therefore we interpret the effects from this model. This model included a main effect of Item-Type ($\hat{\beta } = -0.03$, 95% CI $[-0.05, -0.01]$, $\textit{t}(117) = -3.01$, $\textit{p} = .003$), such that participants increased their looking time to generic items (M = 39, SD = 12) more than child-specific items (M = 33, SD = 14); and a main effect of speaker gender such that participants increased their looking time more for female speakers relative to male speakers (as reported above).

There were also significant interactions between Speaker-Age and speaker-gender ($\hat{\beta } = -0.04$, 95% CI $[-0.06, -0.02]$, $t(117) = -4.19$, $\textit{p} < .001$), such that when the speaker was male, participants increased their target looking more when the speaker was a child (M = 38, SD = 11) than an adult (M = 28, SD = 16), while for female speakers the pattern was reversed (child M = 36, child SD = 11; adult M = 42, adult SD = 11). There was also a significant interaction between Item-Type and speaker-gender ($\hat{\beta } = 0.02$, 95% CI $[0.01, 0.04]$, $\textit{t}(117) = 2.71$, $\textit{p} = .008$), such that for female speakers, participants increased their looking time to generic (M = 39, SD = 11) and child-specific (M = 39, SD = 12) items to similar extents. In contrast, in the male condition, participants increased their looking time to generic items (M = 38, SD = 13) more than child-specific items (M = 28, SD = 14).

These analyses suggest that male adult speech was more difficult to understand in noise (despite leading to slightly higher overall target looking in silence in Experiment 1). We also found that participants increased their target looking more when the target object was generic relative to when it was child-specific. While there were no interactions between Speaker-Age and Item-Type here, interactions with speaker-gender suggest that the pattern of results differed for male and female speakers.

**Table 4:** Experiment 2 growth curve modeling results for female speech condition. Ot1 refers to linear term, Ot2 refers to quadratic term, Ot3 refers to cubic term, Ot4 refers to quartic term

Term	$\hat{\beta }$	95% CI	*t*	$\textit{df}$	*p*
Intercept	0.36	[0.32, 0.40]	19.53	37.43	< .001
Ot1	1.12	[1.09, 1.15]	68.04	83,728.99	< .001
Ot2	-0.68	[-0.71, -0.64]	-41.18	83,728.99	< .001
Ot3	0.22	[0.19, 0.25]	13.37	83,728.99	< .001
Ot4	0.04	[0.01, 0.07]	2.41	83,728.99	.016
Speaker contrast1	-0.03	[-0.03, -0.03]	-18.11	83,735.15	< .001
Type contrast1	0.00	[-0.03, 0.02]	-0.33	22.00	.747
Ot1 $\times$ Speaker contrast1	0.12	[0.09, 0.16]	7.57	83,728.99	< .001
Ot2 $\times$ Speaker contrast1	0.05	[0.02, 0.08]	3.15	83,728.99	.002
Ot3 $\times$ Speaker contrast1	-0.13	[-0.16, -0.10]	-7.81	83,728.99	< .001
Ot4 $\times$ Speaker contrast1	0.10	[0.06, 0.13]	5.80	83,728.99	< .001
Ot1 $\times$ Type contrast1	0.16	[0.13, 0.19]	9.88	83,728.99	< .001
Ot2 $\times$ Type contrast1	-0.01	[-0.04, 0.02]	-0.69	83,728.99	.489
Ot3 $\times$ Type contrast1	-0.05	[-0.08, -0.02]	-3.16	83,728.99	.002
Ot4 $\times$ Type contrast1	0.03	[0.00, 0.06]	1.66	83,728.99	.097
Speaker contrast1 $\times$ Type contrast1	-0.01	[-0.01, 0.00]	-4.27	83,732.36	< .001
Ot1 $\times$ Speaker contrast1 $\times$ Type contrast1	0.04	[0.01, 0.08]	2.66	83,728.99	.008
Ot2 $\times$ Speaker contrast1 $\times$ Type contrast1	0.06	[0.03, 0.09]	3.51	83,728.99	< .001
Ot3 $\times$ Speaker contrast1 $\times$ Type contrast1	-0.06	[-0.09, -0.03]	-3.67	83,728.99	< .001
Ot4 $\times$ Speaker contrast1 $\times$ Type contrast1	0.02	[-0.01, 0.05]	1.18	83,728.99	.238

**Fig. 3:**
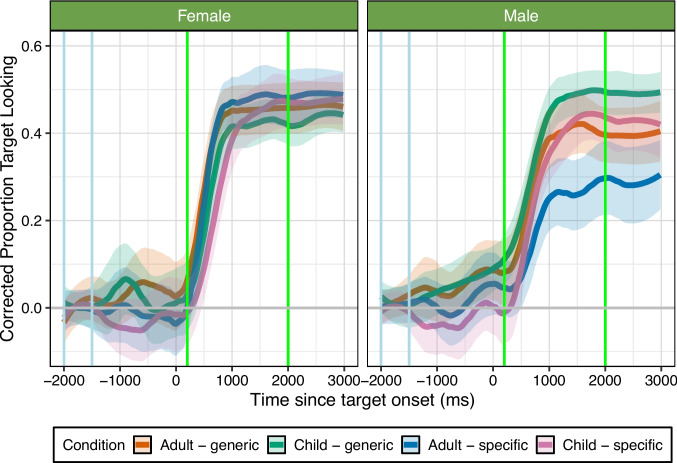
Experiment 2: corrected proportion of looking to the target over time based on condition. Each *line* represents one Speaker-Age and Item-Type condition mean, and *shaded ribbons* show 95% confidence intervals using a normal distribution, smoothed using a span of 0.25. The *x*-axis shows the time since word onset in milliseconds (ms) and the *y*-axis indicates the proportion of target looking; -2000 to -1500 ms (*blue vertical lines*) is the baseline preference window of analysis, target word happened at 0 ms, 200–2000 ms (*green vertical lines*) is the time window for analysis. The *grey horizontal line* indicates 0 change from baseline looking. Overall, this graph shows that participants reached higher peaks when female adults produced speech, while for male speech, participants reached higher peaks for child-produced speech, and for generic items. See Supplementals for looking time graph with overlaid model predictions

For the growth curve model, we followed the same model selection process used in Experiment 1. The model including all four time terms was the best fit (p < .001). Here, too, including speaker gender improved model fit (*p* < .001), and therefore we reran models for female and male speakers separately to improve interpretability.

In the female speaker condition, the model revealed a significant interaction between Speaker-Age and Item-Type, as well as a significant three-way interaction between the linear, quadratic, and cubic time terms and Speaker-Age and Item-Type, see Table 4 for full model output. As demonstrated in Fig. 3, the looking time patterns now differ relative to Experiment 1, participants were faster (linear term) and reached higher peaks (quadratic term) on adult speech relative to child speech, and the sharpness of the peaks varied as a function of Speaker-Age and Item-Type. Here, participants reached higher peaks for adult speech, but exhibited higher peaks for child-specific items when produced by both children and adults.

In the male speaker condition, the model again revealed a significant interaction between Speaker-Age and Item-Type, as well as significant three-way interactions between the linear, quadratic, and quartic terms and Speaker-Age and Item-Type, see Table 5 for full model output. As demonstrated in Fig. 3, participants showed different slopes and peaks across conditions. Specifically, participants actually increased their looking time more in child-produced speech conditions, reaching the highest peak when children produced generic items. In contrast, participants struggled most with looking to the target when it was produced by an adult and it was child-specific. Patterns of increases in target looking were similar for adults producing generic items and children producing child-specific items. Combined with the overall looking analyses, these findings suggest that male adult speech was harder to process in noise, but that background noise may have resulted in adults relying more on their predictions, and that adults are more likely to talk about generic items than child-specific items.

#### Exploratory analysis of self-reported data on experience with children

We analyzed participants’ self-reported frequency of interaction with children as in Experiment 1. Here, participants’ average Experience with Children score was 7.48. Again, we added the Experience with Children Score to our best-fit model, which included Speaker-Age, Item-Type, speaker gender, four time terms, and their interactions as predictors. Then, we ran an ANOVA to compare the last best-fit model with the new one including Experience with Children Score and saw that the addition of this score did not significantly improve model fit (*p* = .679) suggesting that in the presence of pink background noise, the amount of experience with children did not affect participants’ recognition of child-produced speech and child-specific items, see Supplemental Materials for histograms plotting the distribution of these scores as well as additional analyses using a categorical variable of experience.

**Table 5:** Experiment 2 growth curve modeling results for male speech condition. Ot1 refers to linear term, Ot2 refers to quadratic term, Ot3 refers to cubic term, Ot4 refers to quartic term

Term	$\hat{\beta }$	95% CI	*t*	$\textit{df}$	*p*
Intercept	0.30	[0.26, 0.35]	12.39	39.10	< .001
Ot1	1.07	[1.04, 1.11]	64.14	88,228.01	< .001
Ot2	-0.48	[-0.52, -0.45]	-29.01	88,228.01	< .001
Ot3	0.00	[-0.03, 0.04]	0.27	88,228.01	.790
Ot4	0.15	[0.11, 0.18]	8.82	88,228.01	< .001
Speaker contrast1	0.04	[0.04, 0.04]	23.17	88,231.34	< .001
Type contrast1	-0.05	[-0.09, -0.01]	-2.54	22.00	.018
Ot1 $\times$ Speaker contrast1	0.19	[0.16, 0.22]	11.48	88,228.01	< .001
Ot2 $\times$ Speaker contrast1	-0.06	[-0.10, -0.03]	-3.85	88,228.01	< .001
Ot3 $\times$ Speaker contrast1	0.00	[-0.03, 0.03]	0.04	88,228.01	.965
Ot4 $\times$ Speaker contrast1	-0.03	[-0.06, 0.01]	-1.55	88,228.01	.120
Ot1 $\times$ Type contrast1	0.01	[-0.02, 0.04]	0.62	88,228.01	.534
Ot2 $\times$ Type contrast1	0.05	[0.02, 0.09]	3.28	88,228.01	.001
Ot3 $\times$ Type contrast1	0.01	[-0.02, 0.04]	0.56	88,228.01	.575
Ot4 $\times$ Type contrast1	0.01	[-0.03, 0.04]	0.34	88,228.01	.733
Speaker contrast1 $\times$ Type contrast1	0.01	[0.00, 0.01]	4.72	88,230.99	< .001
Ot1 $\times$ Speaker contrast1 $\times$ Type contrast1	0.12	[0.09, 0.16]	7.32	88,228.01	< .001
Ot2 $\times$ Speaker contrast1 $\times$ Type contrast1	-0.08	[-0.12, -0.05]	-5.07	88,228.01	< .001
Ot3 $\times$ Speaker contrast1 $\times$ Type contrast1	0.00	[-0.04, 0.03]	-0.19	88,228.01	.847
Ot4 $\times$ Speaker contrast1 $\times$ Type contrast1	-0.03	[-0.07, 0.00]	-2.04	88,228.01	.042

### Experiment 2 Discussion

Comparing the results of the first two experiments, we can see that adding the pink background noise significantly influenced word recognition, as adults increased their looking time to the target less in the pink noise condition (M = 36, SD = 7.00) compared to Experiment 1 without any background noise (M = 43, SD = 6, *p* < .001). However, this effect was driven by the male speech condition, as in Experiment 2 participants increased their looking time to the target significantly less for male speech (M = 33, SD = 14) than in Experiment 1 (M = 45, SD = 9, $\textit{t} (137.89) = 6.35$, $\textit{p} < .001$), a difference that was not significant for female speech across experiments, $\textit{t} (149.03) = 1.09$, $\textit{p} = .278$.

Comparing across these experiments, we can see that for female speech, background noise actually did not negatively impact the processing of either the adult or child speech. If anything, participants increased their target looking more for adult speech in background noise, though this difference is not significant. For male speech, background noise increased task difficulty overall, but had the largest effect when adults produced child-specific items. First, this suggests that male speech is more masked by background noise than female speech, a finding that has previously been reported in the literature (Brown & Bacon, 2010; McBride, Hodges, & French, 2008; Oh et al., 2022). However, since the biggest “cost” was seen when male adults produced child-specific items, this may also suggest that the presence of pink background noise made it harder for participants to process sentences that were more difficult to predict – male adults talking about child-specific items. In other words, while participants may have been able to rely on predictions to expect male adults to talk about generic items, and children to talk about generic or child-specific items, male adults talking about child-specific items may not have conformed to any predictions. In the next experiment, we examined how background noise that is representative of a child’s natural environment would affect adults’ word recognition; possibly as it might make predicting that children would be talking, or that adults (male or female) would be talking about child-specific things, easier.

## Experiment 3

### Method

#### Participants

Thirty-nine monolingual English speakers (mean age = 20, SD = 1.76) participated in Experiment 3. One additional participant was tested but excluded from the analyses due to not contributing enough data after data cleaning, see below. Participants self-disclosed their race and ethnicity: 28 identified as White, three as Asian, five as Black or African American, and three identified as Other; 36 identified as not Hispanic or Latino, and three Hispanic or Latino.

#### Stimuli

The materials were the same as in Experiment 1. However, this time, the sentence prompts were embedded with real-world background noise. To create real-world background noise clips that simulate an auditory environment where one may hear speech from children or about child-specific things, we used clips from a LENA daylong recording corpus (VanDam, 2018) accessed via Homebank (VanDam et al., 2016). We selected a recording for a target child who was 46 months old, as this was closest to the age of the child speakers used in our experiments. We extracted LENA segmented clips from adult female (FAN), adult male (MAN), and electronic (ELN) segments. We selected 14 clips from each of the three noise groups and ensured that child speech was not present in any of the clips. We created background noise files that included both an electronic clip and an adult clip. Specifically, electronic clips were pseudo-randomly combined with FAN clips to create 48 distinct background sounds for conditions that included father and son target sentences and pseudo-randomly combined them with MAN clips to create 48 distinct background sounds for conditions that included mother and daughter target sentences. Then, each of these combined background noise sound files was pseudo-randomly assigned to one of the target sounds, and their amplitude and length were also matched to the amplitude and length of the target sentences, resulting in a 0dB signal-to-noise ratio (0SNR)^1^. As above, the target word always occurred at 2000-ms post-trial start. Thus, participants who heard female talkers (mother and daughter pair) during the experiment heard the female-produced target sentences embedded in background noise from electronics and MAN clips, while the participants who heard male talkers (father and son pair) heard the male-produced target sentences embedded in background noise from electronics and FAN clips. While this noise itself was not directly relevant to the content of the speech, following prior research on contextual cue effects (Mitchel & Weiss, 2010; Stilp, 2020), we proposed that these sounds taken from children’s homes (specifically the electronic sounds from e.g., children’s songs) could serve as a contextual cue that primes listeners to think of children.

#### Procedure

The procedure was the same as in Experiment 1.

#### Data analysis

We followed the same data analysis pipeline as in Experiment 1.

### Results

In Experiment 3, 60 trials from 12 participants were excluded due to more than 25% track loss. After excluding one participant who contributed only 11 trials in the adult-produced speech condition and therefore did not meet our preregistered inclusion criteria, all participants contributed data for 17 trials in the adult-produced speech condition and 17 in the child-produced speech condition. The average data contribution of all participants was 47 trials, and ranged from 38 to 48.

Using our corrected target-looking procedure, we tested the influence of our counterbalancing decisions on participants’ performance. We saw that the age of the first speaker ($b = -0.01$, 95% CI $[-0.06, 0.04]$, $\textit{t}(36) = -0.31$, $\textit{p} = .757$) did not influence participants’ performance during the task, but their gender did ($b = 0.13$, 95% CI $[0.11, 0.15]$, $\textit{t}(36) = 12.22$, $\textit{p} < .001$) such that the mean increase in target looking for female speakers (M = 36.76, SD = 6) was higher than male speakers (M = 10.50, SD = 7).

Next, overall increases in target looking were analyzed as a function of Speaker-Age, Item-Type, and their interaction. As above, we tested whether including speaker-gender would improve model fit. It did (*p* < .001), so we interpret that model here. This model included an effect of speaker-gender ($\hat{\beta } = 0.13$, 95% CI $[0.11, 0.15]$, $\textit{t}(36) = 12.50$, $\textit{p} < .001$), such that participants increased their looking time more to female speech, as reported above. There were also significant interactions between Speaker-Age and speaker-gender ($\hat{\beta } = -0.05$, 95% CI $[-0.07, -0.03]$, $\textit{t}(108) = -5.03$, $\textit{p} < .001$) and Item-Type and speaker gender ($\hat{\beta } = -0.03$, 95% CI $[-0.05, -0.01]$, $\textit{t}(108) = -3.29$, $\textit{p} = .001$), which are best characterized by a three-way interaction between Speaker-Age, Item-Type and speaker gender ($\hat{\beta } = 0.02$, 95% CI $[0.00, 0.04]$, $\textit{t}(108) = 2.39$, $\textit{p} = .018$).

For female speech, participants increased their target looking more overall for adult speech (M = 42, SD = 14) than child speech (M = 31, SD = 10). They also increased their target looking more for generic (M = 40, SD = 13) than child-specific items (M = 34, SD = 13). For male speech, participants increased their target looking more overall for child speech (M = 15, SD = 14) than adult speech (M = 6, SD = 13), and increased their looking time more for child-specific (M = 14, SD = 12) relative to generic items (M = 7, SD = 15). These patterns were driven by participants not increasing their looking time when male adults produced generic items (M = 0, SD = 13). These results suggest that the presence of real-world background noise further increased challenges in processing male speech, and male adult speech, specifically.

**Table 6:** Experiment 3 growth curve modeling results for female speech condition. Ot1 refers to linear term, Ot2 refers to quadratic term, Ot3 refers to cubic term, Ot4 refers to quartic term

Term	$\hat{\beta }$	95% CI	*t*	$\textit{df}$	*p*
Intercept	0.33	[0.29, 0.38]	15.21	37.93	< .001
Ot1	1.03	[1.00, 1.07]	57.74	74,641.01	< .001
Ot2	-0.61	[-0.65, -0.58]	-34.12	74,641.01	< .001
Ot3	0.23	[0.20, 0.27]	12.88	74,641.01	< .001
Ot4	-0.01	[-0.05, 0.02]	-0.63	74,641.01	.531
Speaker contrast1	-0.05	[-0.06, -0.05]	-27.63	74,645.52	< .001
Type contrast1	-0.03	[-0.06, 0.01]	-1.62	22.00	.120
Ot1 $\times$ Speaker contrast1	0.11	[0.07, 0.14]	5.93	74,641.01	< .001
Ot2 $\times$ Speaker contrast1	-0.04	[-0.07, 0.00]	-2.20	74,641.01	.028
Ot3 $\times$ Speaker contrast1	-0.02	[-0.06, 0.01]	-1.37	74,641.01	.169
Ot4 $\times$ Speaker contrast1	0.07	[0.04, 0.11]	3.95	74,641.01	< .001
Ot1 $\times$ Type contrast1	0.16	[0.12, 0.19]	8.78	74,641.01	< .001
Ot2 $\times$ Type contrast1	-0.07	[-0.10, -0.03]	-3.76	74,641.01	< .001
Ot3 $\times$ Type contrast1	-0.05	[-0.08, -0.01]	-2.69	74,641.01	.007
Ot4 $\times$ Type contrast1	0.04	[0.00, 0.07]	2.03	74,641.01	.042
Speaker contrast1 $\times$ Type contrast1	0.02	[0.02, 0.02]	10.04	74,643.35	< .001
Ot1 $\times$ Speaker contrast1 $\times$ Type contrast1	0.05	[0.02, 0.09]	2.93	74,641.01	.003
Ot2 $\times$ Speaker contrast1 $\times$ Type contrast1	-0.02	[-0.05, 0.02]	-0.99	74,641.01	.324
Ot3 $\times$ Speaker contrast1 $\times$ Type contrast1	-0.03	[-0.06, 0.01]	-1.54	74,641.01	.125
Ot4 $\times$ Speaker contrast1 $\times$ Type contrast1	-0.03	[-0.07, 0.00]	-1.83	74,641.01	.068

**Fig. 4:**
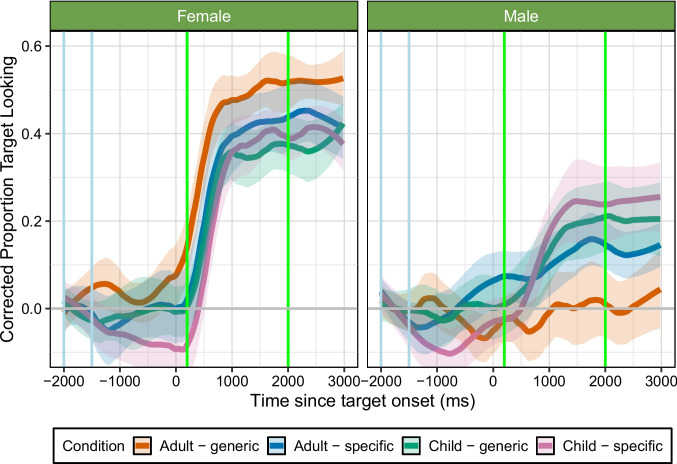
Experiment 3: corrected proportion of looking to the target over time based on condition. Each line represents one Speaker-Age and Item-Type condition mean, and shaded ribbons show 95% confidence intervals using a normal distribution, smoothed using a span of 0.25. The *x*-axis shows the time since word onset in milliseconds (ms) and the *y*-axis indicates the proportion of target looking; -2000 to -1500-ms (*blue vertical lines*) is the baseline preference window of analysis, target word happened at 0 ms, 200–2000 ms (*green vertical lines*) is the time window for analysis. The *grey horizontal line* indicates 0 change from baseline looking. Overall, this graph shows that participants increased their looking time fastest for generic items produced by adults for female speech, and reached the highest peak for child-specific items produced by children for male speech. See Supplementals for looking time graph with overlaid model predictions

In the final step of analysis, we used the growth-curve modeling to look at the pattern of participants’ looking to the target over time in all of the conditions. The model that included the main effects of the Speaker-Age, Item-Type, and interactions with four time terms (linear, quadratic, cubic, and quartic) was a better fit compared to the ones with less time terms (*p* < .001). Here, too, a model that included speaker gender was a significantly better fit for the data (*p* < .001), so we reran the models for female and male speech separately.

For female speech, there was a significant interaction between Speaker-Age and Item-Type, as well as a significant three-way interaction between the linear time term and Speaker-Age and Item-Type. See Table 6 for full model output. Figure 4 shows that, despite the background noise indicating the presence of children, participants still increased their looking time fastest (linear term) when female adults produced generic items. While looking patterns were similar for the remaining three conditions, participants still increased their looking time the least when children produced generic items.

For male speech, there was a significant interaction between Speaker-Age and Item-Type, as well as significant three-way interactions between the quadratic time term and Speaker-Age and Item-Type, see Table 7 for full model output. Figure 4 highlights a similar pattern to Experiment 2, participants’ slope and peak increases in target looking differed as a function of condition. Most notably, participants reached the highest peak (quadratic term) when children produced child-specific items, followed by children producing generic items. They increased target looking least when adults produced generic items, not increasing their looking time at all when these were named, as reflected also in the overall looking time analyses.

#### Exploratory analysis of self-reported data on experience with children

In Experiment 3, participants’ self-reported Experience with Children Score was 5.95. This score was added to our best-fit model that included Speaker-Age, Item-Type, speaker gender, four time terms, and the interaction between them as the predictors. Next, we ran ANOVA to compare the last best-fit model with the new one including Experience with Children Score and saw that the addition of this score did not significantly improve model fit (*p* = .996) suggesting that the amount of experience with children did not affect participants’ recognition of child-produced speech and child-specific items in the presence of real-world background noise, see Supplemental Materials for histograms plotting the distribution of these scores as well as additional analyses using a categorical variable of experience.

### Experiment 3 Discussion

Comparing across experiments, we find that participants increased their target looking less in Experiment 3 (M = 23) than in Experiment 2 (M = 36, $\textit{t} (267.43) = 6.90$, $\textit{p} < .001$), but here, too, this was driven by the male speech condition (Male Exp 2 M = 33; Male Exp 3 M = 11; $\textit{t} (161.91) = 10.17$, $\textit{p} < .001$), as there were no differences in target looking in the female speech condition (Female Exp 2 M = 39; Female Exp 3 M = 37; $\textit{t} (139.51) = 0.94$, $\textit{p} = .351$). Comparing to Experiment 1 reveals the same pattern, an overall difference ($\textit{t} (227.63) = 11.45$, $\textit{p} < .001$) that is driven by the male speech condition ($\textit{t} (132.12) = 18.64$, $\textit{p} < .001$) with a marginal difference in the female speech condition ($\textit{t} (136.83) = 1.89$, $\textit{p} = .061$).

We hypothesized that real-world background noise would help participants predict that children would be speaking, increasing target looking for child speech and possibly specifically child-specific items. We found some evidence for this pattern only in the male speech condition. Compared to pink background noise, which is uninformative, participants who heard male speech in real-world background noise increased their looking time more for child-specific items relative to generic items, when produced by both adults and children. This suggests that participants could have used the real-world background noise to make predictions about what would be talked about. However, this pattern was not evident in the female speech condition, in which the presence of background noise did not have any effect on increases in target looking.

**Table 7:** Experiment 3 growth curve modeling results for male speech condition. Ot1 refers to linear term, Ot2 refers to quadratic term, Ot3 refers to cubic term, Ot4 refers to quartic term

Term	$\hat{\beta }$	95% CI	*t*	$\textit{df}$	*p*
Intercept	0.09	[0.05, 0.14]	4.02	40.47	< .001
Ot1	0.53	[0.49, 0.56]	29.23	85,349.01	< .001
Ot2	-0.09	[-0.13, -0.06]	-5.23	85,349.01	< .001
Ot3	-0.10	[-0.13, -0.06]	-5.33	85,349.01	< .001
Ot4	0.05	[0.02, 0.09]	3.03	85,349.01	.002
Speaker contrast1	0.04	[0.04, 0.05]	23.39	85,353.14	< .001
Type contrast1	0.03	[0.00, 0.07]	1.98	22	.060
Ot1 $\times$ Speaker contrast1	0.27	[0.23, 0.30]	14.87	85,349.01	< .001
Ot2 $\times$ Speaker contrast1	-0.13	[-0.17, -0.10]	-7.34	85,349.01	< .001
Ot3 $\times$ Speaker contrast1	0.00	[-0.04, 0.03]	-0.27	85,349.01	.785
Ot4 $\times$ Speaker contrast1	0.03	[0.00, 0.07]	1.91	85,349.01	.057
Ot1 $\times$ Type contrast1	0.09	[0.06, 0.13]	5.10	85,349.01	< .001
Ot2 $\times$ Type contrast1	-0.04	[-0.08, -0.01]	-2.30	85,349.01	.022
Ot3 $\times$ Type contrast1	-0.01	[-0.04, 0.03]	-0.52	85,349.01	.602
Ot4 $\times$ Type contrast1	0.00	[-0.04, 0.03]	-0.18	85,349.01	.860
Speaker contrast1 $\times$ Type contrast1	-0.02	[-0.03, -0.02]	-12.65	85,349.76	< .001
Ot1 $\times$ Speaker contrast1 $\times$ Type contrast1	0.03	[-0.01, 0.06]	1.59	85,349.01	.112
Ot2 $\times$ Speaker contrast1 $\times$ Type contrast1	-0.05	[-0.08, -0.01]	-2.53	85,349.01	.011
Ot3 $\times$ Speaker contrast1 $\times$ Type contrast1	-0.01	[-0.05, 0.02]	-0.59	85,349.01	.554
Ot4 $\times$ Speaker contrast1 $\times$ Type contrast1	0.03	[0.00, 0.07]	1.94	85,349.01	.052

## Discussion

The aim of this study was to investigate how young adults process a previously understudied type of non-canonical speech: child-produced speech. Since child-produced speech differs from adult-produced speech due to its less canonical pronunciations, greater variability in pitch and duration, and the presence of predictable phonological errors, processing this speech could be more difficult for adults. We examined participants’ word recognition using a two-picture Visual World paradigm across three experiments. We focused on the role of prediction by varying the child-specificity of the target items and manipulating the background noise in which speech was presented: no background noise, pink noise (artificial noise), and real-world noise (noise from children’s homes). Our main research questions focused on how adults processed speech from children, and the role of top-down processing and prediction in comprehending child-produced speech in silence and background noise. We were particularly interested in whether Speaker-Age would interact with Item-Type, as we expected that participants would increase target looking most when the speaker and item matched (i.e., children produced sentences directing to child-specific items).

In Experiment 1, when participants heard adult- and child-produced speech without background noise, we found an interaction between Speaker-Age and Item-Type, such that participants increased their looking time more and faster when adults produced generic items, and least when children produced generic items. These patterns are consistent with previous research (Borovsky & Creel, 2014) suggesting that participants integrated expectations about the speaker and what was being said. This is also in line with research showing that unpredictable or incongruent words in context are more challenging to process (Delong et al., 2011; Schwanenflugel & Shoben, 1985). In Experiment 2, we found different patterns of results for female and male speech. For female speech, participants increased their looking time more and faster for adult relative to child speech, but did not show effects of generic or child-specific items. For male speech, however, participants actually increased their looking time more and faster for child relative to adult speech, and for generic relative to child-specific items. In Experiment 3, we added real-world background noise to the target sentences. Once again, results differed by speaker gender. For female speech, participants increased their target looking more and faster for adult speech, and for generic items. For male speech, participants exhibited the opposite pattern, increasing their target looking more and faster for child speech and child-specific items. Below, we discuss these results in more detail, focusing on two key effects related to speaker gender: (1) different effects of background noise and (2) listener’s use of contextual cues to support word recognition.

### Speaker gender influences masking effects in noise

Starting with female speech, adding background noise resulted in the expected pattern of results: child-produced speech was more difficult to process than adult-produced speech. Participants looked more and faster to the target when it was labeled by an adult female speaker, suggesting that adult-produced speech may be easier for young adult listeners to process, possibly because it is more canonical and/or more familiar to listeners. This pattern was consistent for both the pink background noise and the real-world background noise, suggesting that the type of background noise did not influence the processing of female speech. These results align with prior research on the challenges of processing non-canonical speech. Studies on accented speech, for example, have suggested that non-canonical pronunciations can impair spoken word recognition (Adank et al., 2009; Clarke & Garrett, 2004) and require more cognitive resources during comprehension (Van Engen & Peelle, 2014). While participants may have been able to overcome these challenges in silence, the presence of background noise may have increased the cognitive load, making child speech more difficult to process, a pattern also observed in studies on accented speech in noise (Adank et al., 2009; Van Engen, 2010). However, these effects were small, as direct comparisons of looking time across experiments were not significantly lower for female speech in noise relative to female speech in silence. Whether this small difficulty is due to reduced familiarity or because it also requires more cognitive resources remains an open question for future research.

Comparing the male and female speech condition reveals a different pattern of results. In silence, there were no overall differences in performance between male and female speech. If anything, participants reached slightly higher peaks in the male speech condition relative to the female speech condition, suggesting that the male speakers used here were not, at baseline, more challenging to comprehend. However, when background noise was added, adult male speech became significantly more challenging for listeners than all other speaker types (female adult, female child, male child), across both types of noise. One possible explanation for this pattern is variation in the pitch of the speakers’ voices (see Supplementals for Table showing pitch of stimuli across speakers, and reporting statistical comparisons). Prior research finds that higher-pitched voices (like those of our female and child speakers here) may be easier to understand in noisy environments (Bradlow et al., 1996). In contrast, lower-pitched voices may be more masked by background noise, potentially contributing to the observed difficulties with adult male speech^2^. This pattern was the same in both types of background noise, even though we tried to equate task difficulty across speaker-gender by creating real-world background noise that included different-gender voices, which have been found to increase sensitivity to speech detection in noise (Leibold et al., 2018). Importantly, these effects emerged despite similar transcription accuracy for male and female speech in real-world noise (see Supplemental Materials).

Taken together, these findings suggest that male adult speech (or the male adult speaker used here specifically) is more difficult to comprehend when embedded in background noise. Critically, however, individuals do not exclusively hear speech from female speakers, despite an over-reliance on female speech stimuli in the developmental (Holtz & Papineau, 2024) and adult literature (Strand, 1999). This finding thus highlights the importance of broadening our stimuli to represent a variety of real-world experiences (across speaker gender, age, and background noises), as patterns of results may differ across these variables.

### Top-down use of context supports speech recognition

Building on the observed gender differences in processing speech in noise, we next discuss whether listeners used contextual cues, such as speaker identity and environmental noise, to engage in top-down predictive processing and recognize speech. In Experiment 1, listeners used the congruence between Speaker-Age and Item-Type to facilitate word recognition, showing higher peaks in target looking for speaker–item pairings that aligned with their expectations (e.g., adults naming generic items, children naming child-specific items). This finding suggests that listeners integrate contextual expectations even in silence (see also Van Berkum et al., 2008).

However, in the presence of background noise, the effects of these contextual cues became more nuanced. For female speech, background noise resulted in a main effect of Speaker-Age: participants showed smaller increases in target looking for child speech compared to adult speech. However, background noise did not significantly reduce speech recognition overall. This suggests that in this relatively simple task, and with higher-pitched female voices, listeners were able to separate the target speech from background noise and sustain performance (Calandruccio & Smiljanic, 2012). We had also predicted that real-world background noise might serve as a cue for the presence of children and allow listeners to engage in top-down predictions, particularly when children produced child-specific items. However, when participants listened to female speakers, we found no evidence that real-world background noise facilitated processing of child-produced speech, child-specific items, or child-specific items produced by children. Regardless of noise condition, listeners continued to show longer looking times for adult speech and generic items.

The most notable context effects emerged in the male-speech condition, particularly when comparing Experiments 2 and 3. While there were no differences in increases in target looking for adult and child-produced speech in silence, participants increased their target looking more for child-produced than adult-produced speech in both types of background noise (as discussed above). Crucially, they also showed sensitivity to the predictive value of background noise. In Experiment 2 (pink noise), where background noise was uninformative, listeners looked more to generic items. In contrast, in Experiment 3, when background noise could be used predictively, participants increased their target looking more for child-specific items overall, with the effect being stronger when child-specific items were produced by a child speaker. We interpret this pattern as support for the notion that we connect what is being said to what is happening in the real world, which enables us to make predictions about the upcoming speech (Altmann & Mirković, 2009). The effect of real-world background noise in boosting recognition of child-produced speech and also child-specific items is consistent with literature suggesting that contextual cues can help speech processing (e.g., Bronkhorst, 2000; Van Engen et al., 2012).

These findings suggest that listeners can use real-world background noise to make top-down predictions about upcoming speech. An open question remains as to why participants engaged in predictive processing only for male speech and not for female speech. Female speech showed less susceptibility to masking effects of noise, potentially reducing the need for top-down predictive processing. In contrast, male speech, especially adult male speech, was more susceptible to masking and thus harder to understand in noise. This greater difficulty may have prompted listeners to rely more heavily on external contextual cues, such as real-world background noise, to make predictions. If so, this would suggest that listeners turn to top-down cues more when acoustic information is degraded. In addition, listeners may have a general bias that female adults are already more related to children, and thus real-world background noise did not further boost this. For example, research in implicit behavioral associations finds that females are more strongly associated with the role of “mom” than males are with the role of “dad” (Park et al., 2010) and that social stereotypes influence language processing (Grant et al., 2020). While we might expect participants to then be better at attending to child-specific items when female speakers produce them, it is possible that they were already integrating this information in their processing (despite lower overall increases in target looking), and thus it did not further shift performance. This gender-specific pattern contrasts with findings from earlier research, which sometimes reports no differences in processing male versus female speech (e.g., Brown & Gaskell, 2014). Future work could further investigate this by directly manipulating signal quality with different gender speakers and examining when listeners shift from relying on the speech signal itself to using contextual expectations.

More generally, our results highlight the role of top-down processing in speech comprehension. In top-down processing, we use context, linguistic knowledge, and predictions to understand spoken words, in this case, to make sense of non-canonical speech and speech in noise. These mechanisms in auditory perception are similar to figure-ground organization in vision, where perceptual systems extract relevant information from noisy backgrounds (Peterson & Gibson, 1993). Similar processes underlie auditory segregation: both low-level acoustic features and high-level knowledge (e.g., attention, memory, expectations) contribute to identifying the speech signal (Peterson, 2014). Also, consistent with the idea of “child-directed listening” (Meylan et al., 2023), our findings suggest that listeners may have shifted their expectations when cued by real-world context. In this case, background noise from children’s environments may have primed listeners to expect the presence of children and child-related content. This kind of top-down processing has been found to be beneficial for understanding spoken language, especially under challenging conditions. For example, Hannemann et al. (2007) presented participants with acoustically degraded speech (i.e., unintelligible signals) and found that they used top-down knowledge to improve comprehension. Sohoglu et al. (2012) also explored how listeners use prior knowledge and context in understanding speech and demonstrated that listeners rely on top-down processing for speech perception, especially in degraded auditory signals that are more challenging to understand. However, our results suggest that the degree to which listeners engage in predictive processing may vary depending on both the speech signal and listener biases. This opens the door to future research examining when and why listeners rely on top-down information, particularly in relation to social expectations about speakers (i.e., who tends to talk about what).

### Beyond pitch: interpreting male child speech in noisy environments

While our findings show that listeners might use top-down expectations to integrate contextual cues like background noise and speaker identity, these effects may also be shaped by additional factors. In particular, the question is why there was a bigger cost for processing male child-produced speech in noise, despite it actually being higher in mean pitch relative to the female adult and child speaker (see Supplementals for table showing pitch of stimuli and formant values across speakers, and reporting statistical comparisons). If speakers’ mean pitch predicted intelligibility in noise, then we would have expected male child speech to be the easiest to process. Since this was not the case, it seems unlikely that the observed difficulty can be fully explained by the extent to which background noise masked each speaker.

One possibility is that speaker gender interacted with presentation order, such that participants who heard male adult speech first were primed to not expect children, however if we test for an interaction between speaker gender and counterbalancing order, the interaction is not significant in Experiment 2 ($\textit{t}(37) = 0.87$, $\textit{p} = .390$) or Experiment 3 ($\textit{t}(34) = 1.83$, $\textit{p} = .075$). It is also possible that participants are able to use information in addition to fundamental frequency to estimate a speaker’s gender, even for children. In fact, Barreda and Assmann (2021) found that listeners can distinguish between male and female child voices even when acoustic properties do not differ between them. This aligns with findings from Koenig (2018), which found that adults use both prescriptive and descriptive gender stereotypes about children, indicating that gendered expectations may influence how listeners interpret speech beyond acoustic cues. To probe whether stereotypes are playing a role in processing in this task, future research should vary the type of background noise (e.g. sports bar vs. children’s home environments) and could collect information about participants’ stereotypes or essentialist views to test whether individuals who are more likely to implicitly link females with the role of “mom” also perform differently in processing speech from female speakers about child-specific items.

### Individual and developmental differences in processing child speech

While our findings emphasize the role of top-down expectations and social biases, individual differences in listener experience and developmental stage may also shape speech processing, especially in noisy conditions or processing non-canonical speech. As an exploratory analysis, based on previous studies suggesting that having prior information or experience about the parameters of a signal can increase its detectability (e.g., Wiley, 2017), we tested whether participants’ previous experience with children affected their performance. Across all three experiments, participants’ self-reported frequency of interaction with children did not improve our best model fit, suggesting that the amount of participants’ experience with children did not influence their performance. This finding is in line with Yu et al. (2023); who had found that only experience with a specific child’s speech (i.e. their own child) increased intelligibility. However, earlier studies on native listeners’ comprehension of accented speech suggested that prior experience improves perceptual accuracy (e.g., Clarke & Garrett, 2004). As noted above, it is possible that the child speakers used in this experiment were not particularly challenging, and thus experience is not necessary for relatively easy processing. We might expect different results for children who are diagnosed with speech impediments or language delays. Another possibility is that some listeners may rely more on prior experience than others. For example, listening in a second language may be more challenging overall, and particularly so in noisy environments (Rogers et al., 2006) or for non-canonical speech. Testing second language listeners with and without experience with child-produced speech may allow for insight into whether prior experience is more important for some listeners than others.

Lastly, an open question is how this pattern of results would extend to developmental populations. Previous research suggests that toddlers comprehend adult-produced speech better than that of same-age peers (Cooper et al., 2018). However, previous research does not consider the role of experience with speech produced by other children, or with processing adult- and child-produced speech in noise. Many children have siblings or attend daycare and, consequently, some children could have more experience with child-produced speech than others. Similarly, children’s environments often include background noise, whether that be a busy daycare classroom, or background noise in homes like that used in Experiment 3. We look forward to future research exploring children’s ability to process and recognize child-produced speech in silence as well as in the presence of real-world background noise.

### Conclusion

Taken together, we found that child-produced speech is not generally more challenging to process. Instead, challenges arise when comparing child-produced speech to female adult-produced speech in noise. Within and across experiments, we also manipulated Item-Type and background noise, finding that listeners *can* consider who is speaking when making predictions about what they will say, but they do not always use this information to improve processing. Specifically, our findings suggest that background noise may improve processing by allowing listeners to make predictions (i.e., real-world noise), but that participants may only do this under very challenging conditions, such as male speech. While unplanned, a primary take-away from this study is that the field needs to use representative stimuli, across different categories of speakers, as results may not generalize between male and female speakers, or child and adult speakers. Together, these patterns of results inform our theories of speech perception, highlighting the complicated interplay between the characteristics of speech and speaker, environmental conditions, and listener expectations in speech processing.

## Supplementary Information

Below is the link to the electronic supplementary material.

Supplementary file 1 (pdf 261 KB)

## Data Availability

All data and materials used to generate this manuscript are readily available on OSF: https://osf.io/ewphv/

## Footnotes

1 We conducted a norming study to ensure that male and female speech in real world background noise was similarly intelligible (as measured by transcription accuracy), and found that it was, see Supplemental Materials.

2 See Supplementals for an analysis of speaker’s Formant values

## Code availability

The code used to analyze the data and generate this manuscript is available on OSF: https://osf.io/ewphv/
