# Supplementary material for: Decoding child speech in silence and noise: The type of background noise shapes adults’ processing
Source: Atten Percept Psychophys. 2025 Dec 4;88(1):30. doi: 10.3758/s13414-025-03194-4 (PMC12678573; doi:10.3758/s13414-025-03194-4)
Supplement: Supplementary file 1 — (pdf 261 KB) [file 13414_2025_3194_MOESM1_ESM.pdf]

Supplemental Information for Decoding child speech in silence and noise: the type of background noise shapes adult's processing

Supplemental Information for Decoding child speech in silence and noise: the type of background noise shapes adult's processing

Supplemental Materials Overview

These supplemental materials include the information for: 1) the pitch of the stimuli (with no background noise) for all four speakers and their comparisons, 2) the first (F1) and second (F2) formant values and their ratio for the central vowels embedded in the target words of the stimuli (with no background noise) for all four speakers and their comparisons, 3) a norming study we did to make sure participants could hear bth male and female speakers equally well in the real-world background noise condition, 4) the distribution of participants' self-reported data on experience with children and new categorical analysis using these ratings and 5) graphs for all experiments with the predicted model values overlaid on top of the graphs.

Pitch of the stimuli

Table 1

Pitch and speaker characteristics of stimuli used in study (with no background noise). The first column shows if the speaker was an adult or a child, the second column shows the speaker gender (male or female), the third and fourth columns include the mean pitch and SDs (respectively) of that speakers' sound files

speaker_age	speaker_gender	mean	sd
adult	female	223.94	18.01
adult	male	134.15	10.78
child	female	244.68	24.29
child	male	279.24	25.18

Table 1 shows average pitch for each speaker used in Experiment 1 (with no background noise). We tested whether mean pitch varied as a function of speaker. A two-way ANOVA revealed significant main effects of speaker age ($p < .001$), such that the mean pitch of children (M = 261.96, SD = 30.07) was higher than adults (M = 180.78, SD = 47.67). There was also a main effect of speaker gender ($p < .001$), such that female speakers had significantly higher pitch (M = 233.70, SD = 23.44) than male speakers (M = 205.22, SD = 75.71). The interaction between speaker age and speaker gender was also significant ($p < .001$) such that the effect of speaker-age on pitch was more pronounced for male speakers than for female speakers: child male speakers had the highest pitch values, while adult male speakers had the lowest.

Formant Values of the stimuli

Table 2

Formant values and speaker characteristics of stimuli used in study (with no background noise). The first column shows if the speaker was an adult or a child, the second column shows the speaker gender (male or female), the third and fourth columns include the mean and SDs (respectively) of F1 for each speakers' sound files. The fourth and Fith columns show the same information for F2 values. The last column shows the mean F1/F2 ratio for each speaker.

speaker_age	speaker_gender	mean_F1	sd_F1	mean_F2	sd_F2	mean_ratio
adult	female	744.12	265.16	1782.18	503.24	0.47
adult	male	545.17	115.95	1571.63	585.47	0.41
child	female	732.48	148.82	1796.27	577.35	0.44
child	male	726.07	214.07	2038.52	780.69	0.42

The first (F1) and second (F2) formant values as well as the F1/F2 ratio for the central vowels embedded in the target words of the stimuli (with no background noise) were

extracted at the temporal midpoint of each vowel for all four speakers. Table 2 shows the average F1 and F2 for each speaker. We used two-way ANOVAs to examine whether F1 and F2 formant values varied as a function of speaker age and gender and their interaction. For F1 values, we found a significant effect of speaker age ($p = .043$), such that children had higher F1 values ($M = 729.27$, $SD = 182.41$) than adults ($M = 648.47$, $SD = 228.57$). There was also a significant main effect of speaker gender ($p = .008$), such that female speakers had higher F1 ($M = 738.64$, $SD = 216.29$) than male speakers ($M = 633.77$, $SD = 192.43$). The interaction between speaker age and gender was also significant ($p = .016$), showing a larger gender-based difference in adults: adult females had higher F1 ($M = 744.12$, $SD = 265.16$) than adult males ($M = 545.17$, $SD = 115.95$), whereas the two child speakers showed similar F1 values.

For F2 values, there were no significant main effects of speaker age ($p = .058$) or speaker gender ($p = .955$), or their interaction ($p = .070$). Numerically, child male speakers had the highest F2 values ($M = 2{,}038.52$), but this difference was not significant.

Additionally, the F1/F2 ratio showed no significant effects of speaker age ($p = .858$), speaker gender ($p = .251$), or their interaction ($p = .641$). Together, these results suggest that the observed intelligibility costs for male child speech in noise are unlikely to be driven by differences in formants.

Stimuli norming study

For creating the real-world background noise in the third experiment, we used a combination of female speech and electronic noise to make the background noise for target sentences produced by male speakers, and combined male speech and electronic noise to create the background noise for target sentences produced by female speakers. After creating the stimuli, we felt that male speakers were harder to hear compared to female speakers. Therefore, we conducted a norming study where 20 participants from the university subject

pool transcribed the stimuli produced by either male or female speakers to check whether the male speech condition in 0SNR was in fact more challenging. Their transcriptions were reviewed and marked as accurate if they matched the target sentence, with a focus on the target word. Then, we analyzed their transcription accuracy and found that their accuracy did not significantly differ by speaker ($t(679.22) = -1.25$, $p = .212$), with participants transcribing male speakers with 50% accuracy and female speakers with 46% accuracy. In other words, despite initial impressions that male speakers were harder to hear, participants were equally accurate in transcribing sentences produced by both male and female speakers. We proceeded with the experiment reported in the manuscript, which did reveal differences as a function of speaker gender, despite the comprehension task arguably being simpler than the transcription task, as during the comprehension task participants saw only two options they could look at, compared to the transcription task which allowed participants to type any possible word. At the same time, the transcription task was not time limited, and we did not collect participants' reaction times, so they may have spent additional time processing the speech before typing their response. While this was just a small norming study, this suggests a potential asymmetry between online speech processing and transcription.

Exploratory Analysis of Self-Reported Data on Experience with Children

In our exploratory analyses, we examined whether participants' self-reported experience with children influenced target looking behavior. Initially, we modeled the total score of experience as a continuous predictor but found no significant improvement in model fit across any experiment. The distribution of their total score in all three experiments is shown in Figure 1.

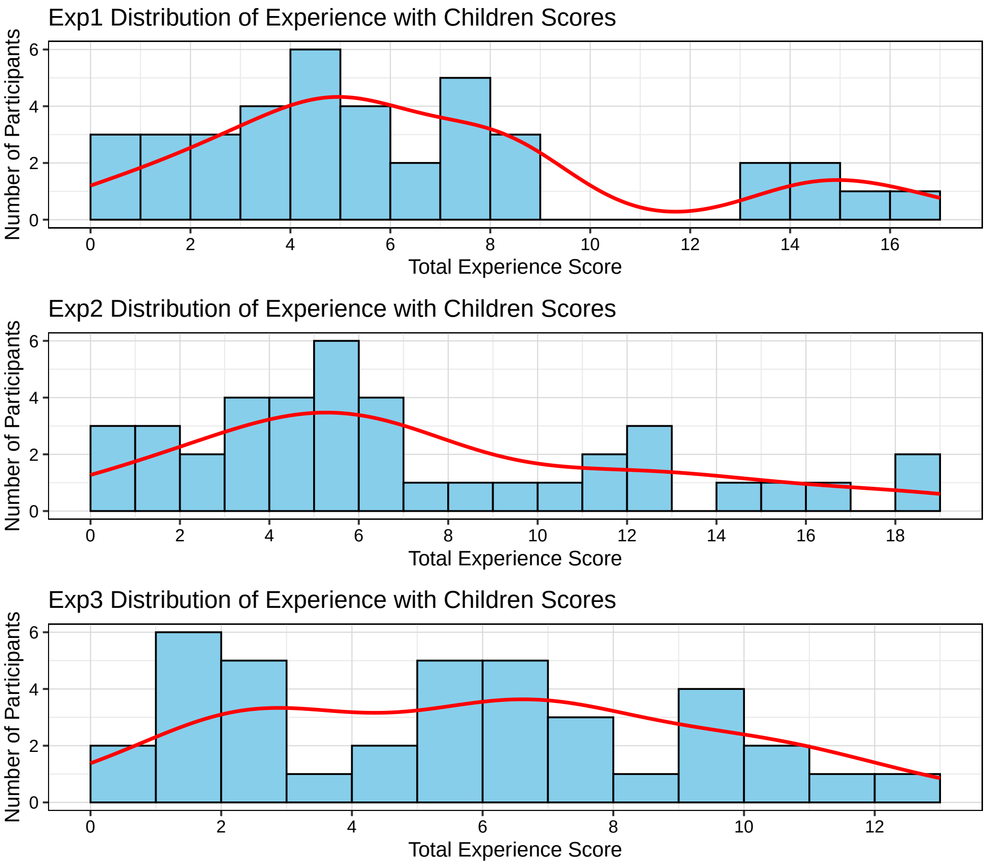

Figure 1. Distribution of participants' experience with children across all three experiments. The x-axis shows the total score of participants' experience with children of different ages. The y axis indicates the number of participants reporting each score.

To further explore whether a different way of categorizing their experience would lead to different effects, in each experiment, we categorized participants into three groups based on their total experience scores: 1) *none*: no reported experience, 2) *some*: below average, and 3) *more*: at or above the average. We then reran our models using this categorical variable and compared them to the original best fit models without this factor using ANOVA (previous best fit models included speaker-age, item-type, speaker-gender, four time-terms, and the interaction between them as the predictors). Again, we found that adding the categorical experience variable did not significantly improve model fit in Experiment 1 ($p = .215$), Experiment 2 ($p = .758$) or Experiment 3 also, ($p = .837$). This suggests that

participants' level of experience with children, even when categorized in this way, did not meaningfully account for variability in their target looking behavior across experiments.

Graphs with predicted model outputs

The looking time graphs in the manusript include 95% CIs, but in order to show the entire looking trajectory (from trial start through the 5000ms), we were unable to overlay the model's predicted values. In this section, we include graphs for just the analyzed portion (from 200-2000ms post naming), plotting standard error ranges with overlayed model estimates.

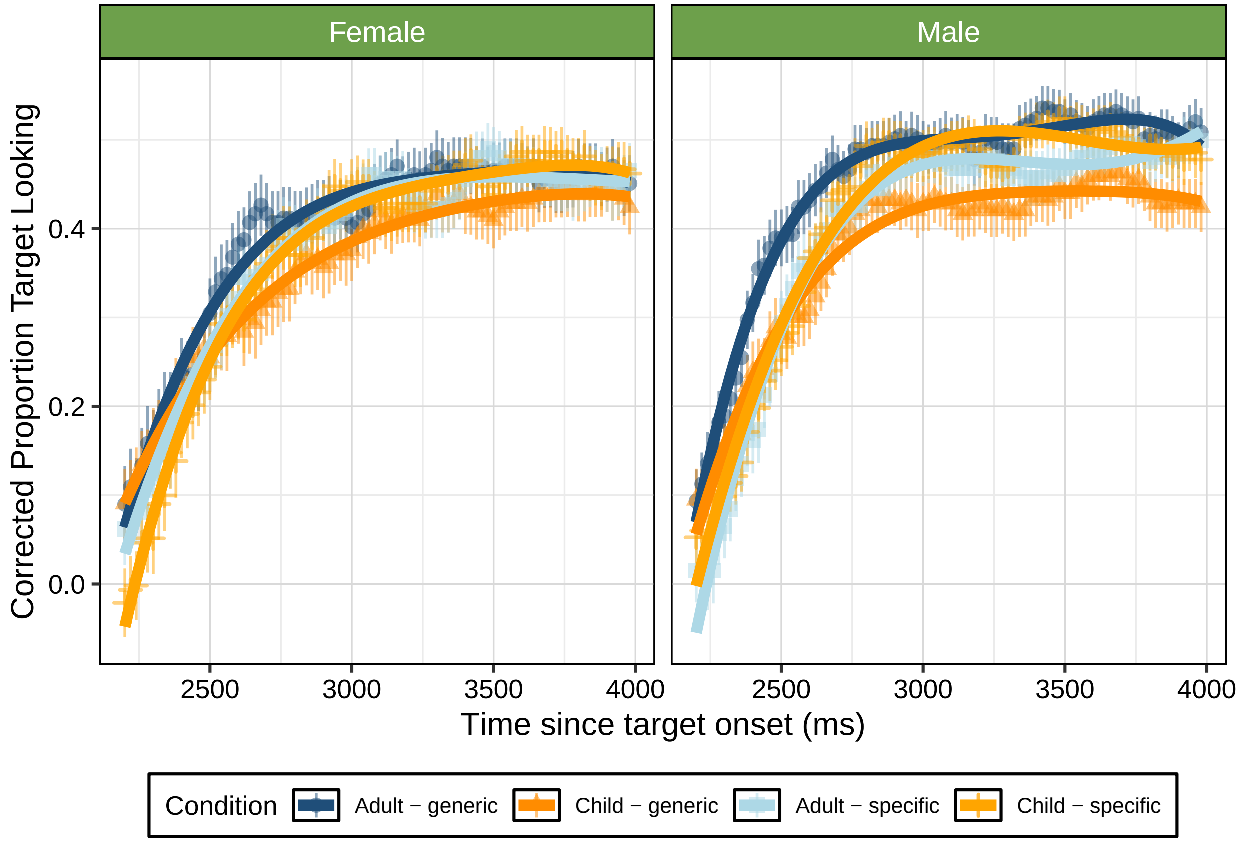

Figure 2. Experiment 1: predicted corrected proportion of looking to the target over time based on condition, pointranges indicate standard error. The x-axis shows the time since word onset in milliseconds (ms) and the y-axis indicates the proportion of target looking.

Figure 2 shows the predicted model values of corrected proportion of looking to the target over time on top of the data points for Experiment 1. Figure 3 and Figure 4 show the predicted values for Experiments 2 and 3, respectively.

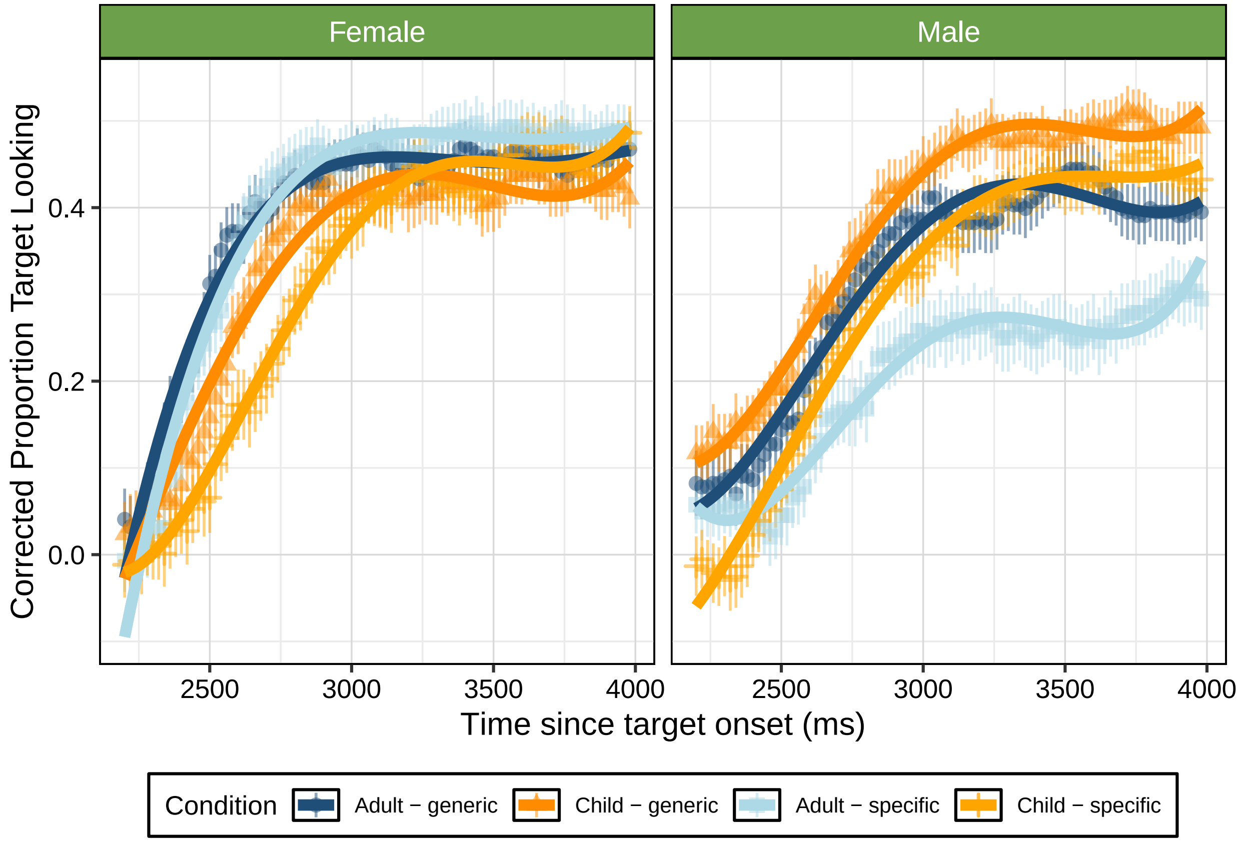

Figure 3. Experiment 2: predicted corrected proportion of looking to the target over time based on condition, pointranges indicate standard error. The x-axis shows the time since word onset in milliseconds (ms) and the y-axis indicates the proportion of target looking.

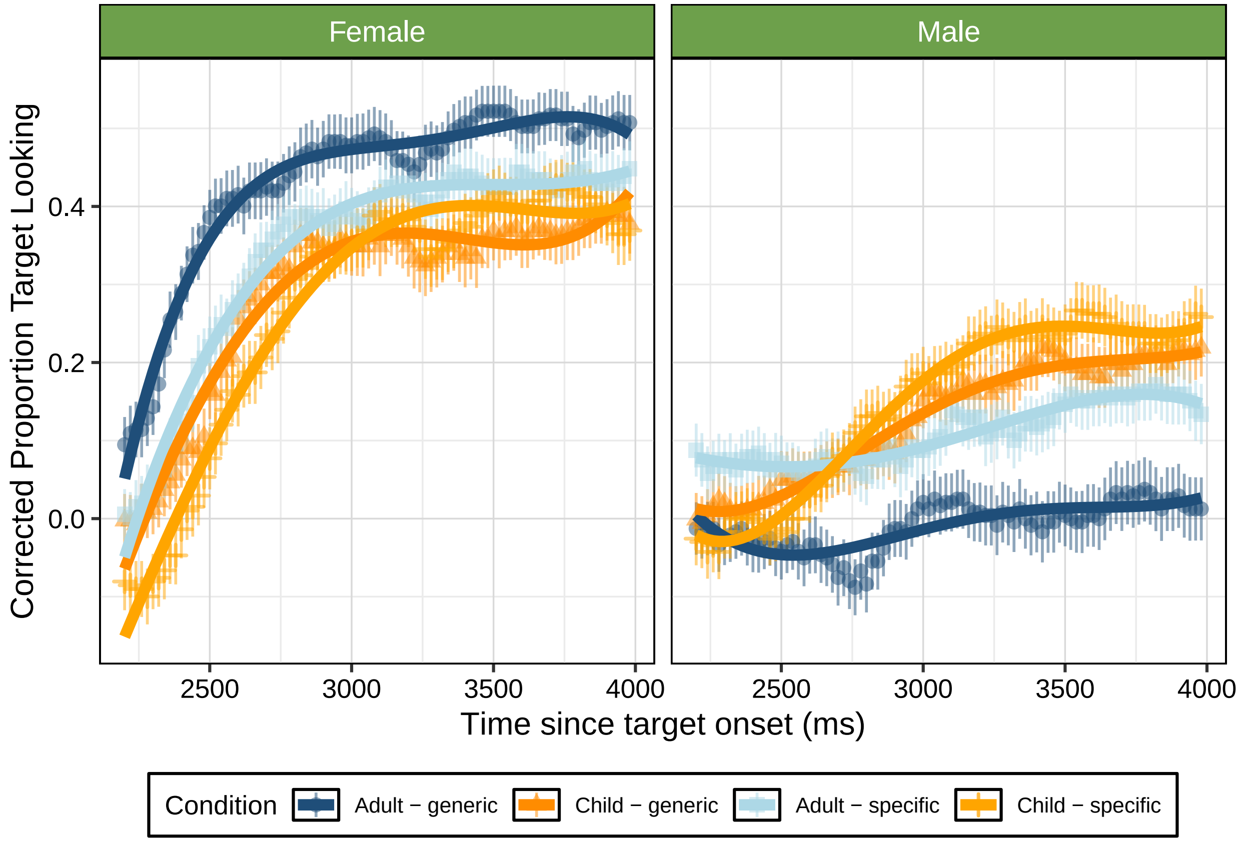

Figure 4. Experiment 3: predicted corrected proportion of looking to the target over time based on condition, pointranges indicate standard error. The x-axis shows the time since word onset in milliseconds (ms) and the y-axis indicates the proportion of target looking.
